# What are the barriers and facilitators to community handwashing with water and soap? A systematic review

**DOI:** 10.1371/journal.pgph.0001720

**Published:** 2023-04-19

**Authors:** Obidimma Ezezika, Jennifer Heng, Kishif Fatima, Ayman Mohamed, Kathryn Barrett

**Affiliations:** 1 Faculty of Health Sciences, University of Western Ontario, London, Ontario, Canada; 2 Department of Health and Society, University of Toronto Scarborough, Scarborough, Canada; 3 African Centre for Innovation & Leadership Development, Abuja, Nigeria; 4 University of Toronto Scarborough Library, Scarborough, Canada; UP Manila: University of the Philippines Manila, PHILIPPINES

## Abstract

Handwashing with water and soap (HWWS) is an effective method of cleaning and disinfecting the surface of the hands. HWWS is effective in infection control and prevention transmission, such as in Severe Acute Respiratory Syndrome Coronavirus 2 (SARS-CoV-2). However, rates of handwashing compliance vary globally. This systematic review aimed to identify the barriers and facilitators to community HWWS globally. We conducted a comprehensive search strategy in OVID Medline, OVID Embase, Web of Science Core Collection, and Scopus using keywords and subject headings related to handwashing. Studies were excluded if they reported hand hygiene among healthcare or food service workers, considered the use of alcohol rubs, or involved an intervention in a healthcare or food preparation setting. The quality of eligible studies was assessed using the Mixed Methods Appraisal Tool, and data were extracted from the articles and analyzed using the Theoretical Domains Framework and inductive thematic analysis. The search strategy yielded a total of 11,696 studies, and 46 studies met the eligibility criteria. Study dates ranged from 2003 to 2020 and included 26 countries; the most frequently represented were Bangladesh, India, and Kenya. A total of 21 barriers and 23 facilitators to HWWS were identified and organized into the Theoretical Domains Framework. The most frequently cited domains were environmental context and resources, goals, and knowledge. Nine themes emerged from these barriers and facilitators: resource availability, cost and affordability, handwash station design and infrastructure, accessibility, gender roles, champions, health promotion, time management, and knowledge, beliefs, and behaviors. This review uncovered multiple barriers and facilitators around a determinant framework to observe and create an in-depth, multidimensional image of a community-based hand hygiene situation. New comprehensive interventions and implementation strategies can be developed using the findings to target the contextual barriers and facilitators to improve and increase HWWS rates. Stakeholders (i.e., practitioners, researchers, policymakers) can use the findings to revise, design, or evaluate new or existing projects, interventions, and policies to improve HWWS.

**Registration:** A protocol for this systematic review was developed and uploaded onto the PROSPERO—International prospective register of systematic reviews database (Registration number: CRD42020221210).

## Introduction

Handwashing has become an effective infection control mechanism and preventative measure against contracting SARS-CoV2 [1]. Proper hand hygiene is a cost-effective method for controlling infectious diseases, but despite hand hygiene promotion and increased public awareness, hand hygiene compliance rates remain low and hard to change [2]. Specifically, low compliance rates are evident after the use of public restrooms [2] and before and after food handling and eating [3]. Despite the benefits of proper hand hygiene, compliance rates remain low in both clinical and general settings.

Over the last few years, several systematic reviews have addressed handwashing practices [4–12]. These studies have focused on hand hygiene compliance in healthcare settings [4], behavioral determinants of handwashing in domestic settings [5–10], the association between diarrheal disease and handwashing [4–6, 9–12], and hand hygiene promotion using psychosocial theory [7–9]. While systematic reviews related to handwashing exist specifically around determinants of HWWS [5, 7, 8], these reviews have either focused on domestic settings [5], approaches to improve handwashing behavior in low and middle-income countries [7], or the effectiveness of handwashing practices [8]. However, to our knowledge, there are no systematic reviews on the barriers and facilitators to community HWWS globally, organized according to a determinant implementation science framework.

This systematic review aimed to answer the following question: What are the barriers and facilitators to community HWWS, globally? The hope of this study is that these findings will provide a more structured organization of the barriers and facilitators to HWWS implementation and help support the generation of hypotheses for improving community-based HWWS.

## Methods

### Search strategy

We conducted a comprehensive search in OVID Medline, OVID Embase, Web of Science Core Collection, and Scopus according to a search strategy developed by an academic health sciences librarian (KB), with input from the research team. The search was structured around three main concepts: handwashing, communicable disease control, and implementation science. These three concepts were represented using search terms such as (1) hand hygiene, hand disinfection, hand washing, hand cleaning, and soap, (2) communicable diseases, disease, illness, sick(ness), outbreak, epidemics, pandemics, infection, transmission, prevention, control, sanitation, and (3) implementation, adoption, intervention, program, model, or terms related to barriers and facilitators, such as behavior, knowledge, attitude, motivation, belief, promotion, efficacy, and policy. We executed the search on June 11, 2020. The results were limited to English-language journal articles involving human subjects, and a publication date limit of 1970 to 2020 was applied. The rationale for the cut-off year of 1970 was that it was before the first official guideline for handwashing and hospital infection control was published by the Centers for Disease Control and Prevention (CDC) in 1985 [13]. This guideline was based on a nationwide study in 1974 by the CDC in the United States on nosocomial infectious disease control [14]. Therefore, the year limit of 1970 captures hand hygiene implementation just before the nationwide CDC study. We provide the search strategies used in each database in S1 File.

### Eligibility criteria and study selection

To be included in the review, studies needed to be English-language primary research articles published in an academic journal between 1970 and 2020. Each study needed to meet the following characteristics: reporting on a hand hygiene or handwashing intervention using soap and water as a means of preventing infectious disease, taking place within a community or school environment, addressing hand hygiene practice, and identifying at least one barrier or facilitator to HWWS. The study participants could be of any age and live in any geographic area.

We excluded articles if they did not discuss HWWS (Table 1). Studies were also excluded if they reported hand hygiene among healthcare or food service workers, considered the use of alcohol rubs, or involved an intervention that took place in a healthcare or food preparation setting.

**Table 1:** Selection inclusion and exclusion criteria for the preliminary analyses of title and abstract screening and full-text review.

Selection Criteria	Inclusion Criteria	Exclusion Criteria
*Publication Characteristics*		
**Language**	English	All languages except English
**Publication Type**	Scholarly journal articles	All publications that are not scholarly journal articles
**Study Type**	Primary (research)	Secondary (review)
**Publication Date**	1970–2020	Articles published before 1970 or after 2020
*Study Characteristics*		
**Issue**	Prevention of communicable or infectious disease	Food safety, preparation, and handling
**Setting**	Community or school setting	Healthcare settings and facilities
		The food industry (including restaurants)
**Population**	Community members or students of any age	Healthcare providers
		Workers in the food or restaurant industry
**Intervention**	Hand hygiene intervention, program, or campaign using soap and water alone	Any intervention that does not involve hand hygiene or hand washing
	Hand hygiene may refer to hand washing, hand disinfection, or hand cleaning	Hand hygiene interventions that do not use soap and water alone (e.g., hand sanitizer or alcohol-based products)
**Outcome**	Discussion of the barriers and facilitators to the implementation, adoption, or scaling up of the hand hygiene intervention	No discussion of the barriers and facilitators to implementation, adoption, or scaling up of the hand hygiene intervention

**Fig 1:**
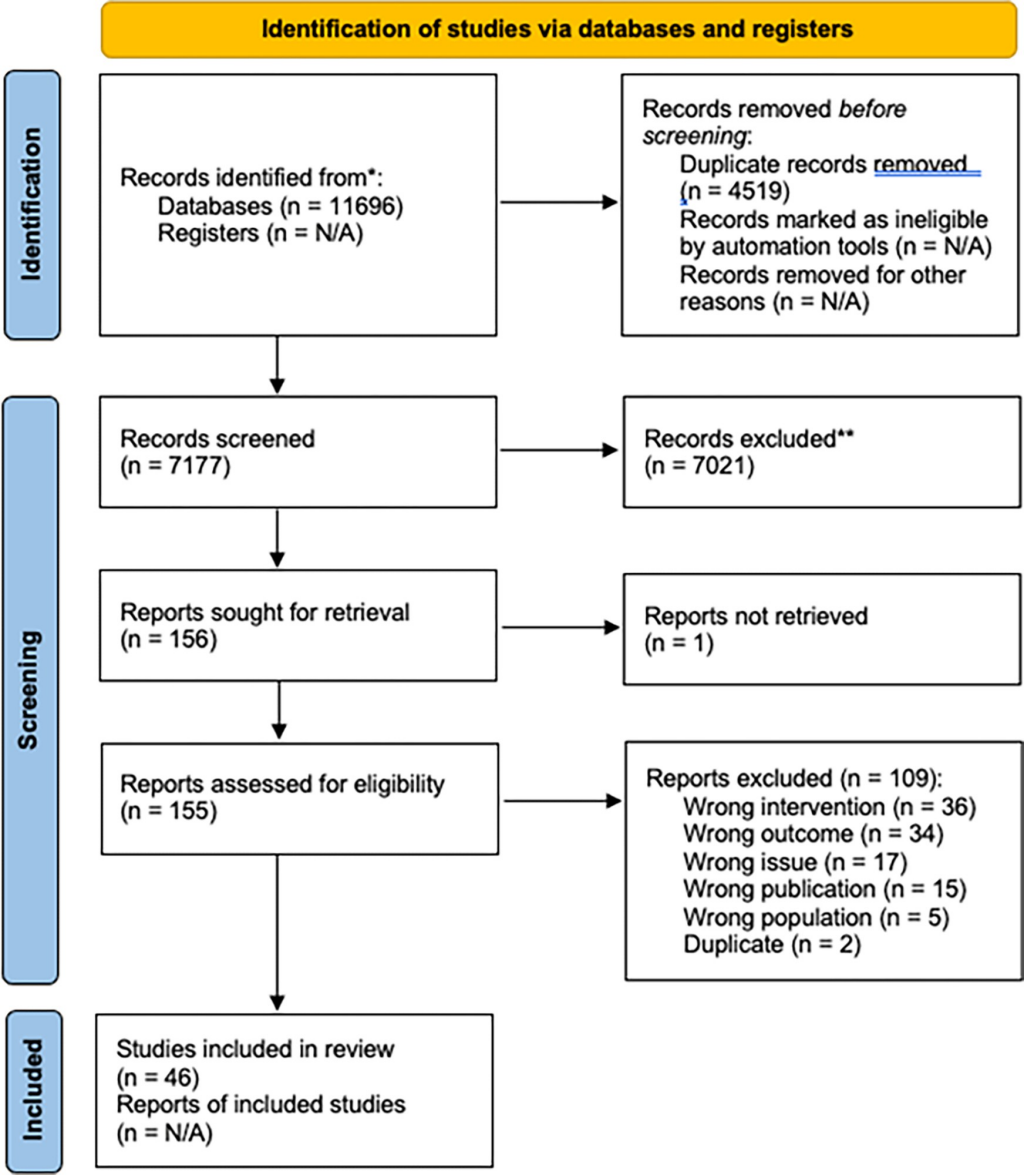


The Preferred Reporting Items for Systematic Reviews and Meta-analysis (PRISMA) framework was used to report the systematic review, as illustrated in S2 File; Covidence, a systematic review management tool, was used to screen studies and generate a PRISMA flow diagram (Fig 1). Article titles and abstracts were screened independently by two reviewers (JH and AM), and conflicts were resolved by a third party (KB). The full texts of the publications were then reviewed independently by two reviewers (JH and KF) for eligibility, and conflicts were resolved by a third party (KB).

### Data extraction and analyses

Data extraction began with two reviewers (JH and KF) independently pilot-testing the data extraction instrument using three eligible articles. Discrepancies in consensus were resolved through a discussion facilitated by OE. Following pilot testing, the same two reviewers independently extracted and agreed on the study characteristics, barriers, and facilitators for all eligible articles. OE checked and resolved any discrepancies. The extracted study characteristics for the 46 articles are provided in S3 File.

After data extraction, similar barriers and facilitators were aggregated under new themes, alongside one chosen representative text excerpt from an article to provide context. After the extraction was finalized, JH and KF independently pilot-tested coding for three aggregated barriers and facilitators using the TDF, with a consensus reached thereafter; then, they completed the TDF coding for the remaining articles and reached a consensus on the coding. OE resolved discrepancies between the individual coding results from the TDF, meeting with KF and JH to adjudicate differences in their coding, and created a consensus template. The coding criteria were based on the definition of the domain and corresponding constructs.

### Quality assessment

The quality of the 46 articles included in the review was assessed using the Mixed Methods Appraisal Tool (MMAT). Forty-five articles passed the MMAT assessment. One article did not pass the appraisal; in this case, the first two screening questions of the MMAT, regarding the identification of a clear research objective were answered with “Can’t Tell.” However, we decided that the article would be accepted because it provided valuable information and context for the barriers and facilitators that it identified. The MMAT assessment results are provided in S4 File.

## Results

From the OVID Medline, OVID Embase, Web of Science Core Collection, and Scopus databases, we retrieved 11,696 studies, and following de-duplication, we screened 7,177 study references and assessed the full text of 156 documents. Forty-six publications met our inclusion criteria, and their data were extracted. Authors reached consensus on eligibility of all the included studies. S3 File summarizes the forty-six studies, including important study elements, study design methods, participants, and study objectives.

### Type of study/methods

Of the 46 studies, 31 were interventional and 15 were non-interventional. An interventional study is defined as one in which the researchers take an active role by intervening with variables that affect a particular outcome to collect and analyze the data (e.g., implementing a health promotion program). A non-interventional study is defined as a study in which the researcher takes a passive role and associations between variables are observed within a population (e.g., observing the level of knowledge of infectious diseases in relation to handwashing behaviors).

The 31 interventional studies included five different study types. Most studies were quantitative and belonged to three types—namely, quantitative descriptive studies (*n* = 2), quantitative non-randomized studies (*n* = 3), and quantitative randomized control trials (*n* = 10). The remaining studies represented the other two types—namely, qualitative (*n* = 9) and mixed-methods studies (*n* = 7). Furthermore, the 31 interventional studies employed a variety of study methods (where the *n* of studies is not exclusive to one method), including surveys (*n* = 9), focus groups (*n* = 8), interviews (*n* = 14), quantitative observation (*n* = 16), and qualitative observation (*n* = 6).

The 15 non-interventional studies included the following four types of studies: qualitative (*n* = 8), quantitative descriptive (*n* = 1), quantitative non-randomized (n = 2), and mixed-methods studies (*n* = 4). Moreover, these studies employed a variety of study methods (where the *n* of studies is not exclusive to one method), including surveys (*n* = 7), focus groups (*n* = 6), interviews (*n* = 10), quantitative observation (*n* = 1), and qualitative observation (*n* = 6).

### Year of publication

Although the search strategy included studies published from 1970 onwards, the oldest study included in the review was published in 2003 (*n* = 1). There were only a few studies published in the subsequent years of 2005 (*n* = 1), 2008 (*n* = 1), 2009 (*n* = 1), 2010 (*n* = 1), and 2011 (*n* = 1). There were more studies in 2012 (*n* = 4) and onwards, and at least two studies that were published thereafter in 2013 (*n* = 5), 2014 (*n* = 2), 2015 (*n* = 3), 2016 (*n* = 4), 2017 (*n* = 5), and 2018 (*n* = 5). Given the specific interest in the start of the SARS-CoV2 pandemic in 2019, it is noteworthy that just under a quarter of the studies (23.9%) were published in 2019 (*n* = 6) and 2020 (*n* = 5).

### Participants

Of the 46 studies, 18 involved participants from various households. Several key events of HWWS (i.e., after defecation, before food preparation, and before eating) occurred within the home. Ten studies were conducted on primary school students only, and 12 were conducted with primary school students, in addition to the teachers, school authorities, and family members of the students. In four studies, women in general and those who were mothers or caregivers of children were selected as participants, based on women’s gender roles. One study selected participants from among university students and staff. Another study selected Water, Sanitation, and Hygiene (WASH) stakeholders as participants for key informant interviews.

### Countries

Of the 46 studies, 26 countries were geographically represented as the study setting. The three most common countries in which studies took place were Bangladesh (*n* = 7), India (*n* = 6), and Kenya (*n* = 4). Three studies each were conducted in South Africa (*n* = 3), United Kindgom (*n* = 3), and Zambia (*n* = 3). Two studies each were conducted in Uganda (*n* = 2) and the United States (*n* = 2). One study was conducted in each of the following countries: Australia, Democratic Republic of the Congo, Egypt, Ghana, Iraq, Malawi, Mali, Myanmar, Nepal, South Korea, Tanzania, Thailand, and Vietnam. Furthermore, there were two studies that occurred in multiple countries, with one conducted in Nepal, Pakistan, and the Philippines (*n* = 1) and the other conducted in Thailand, Ethiopia, and Kenya (*n* = 1). In addition, one study was not conducted in a physical region (*n* = 1) because it was non-interventional and based on interviews with stakeholders on hand hygiene with expertise in Africa, Asia, and Central/South America and the Caribbean.

### Study objectives of the selected papers

Twenty-nine studies focused specifically on the outcomes of hand hygiene interventions that involved HWWS. More specifically, these interventions (i.e., installation of handwashing materials and technology, health promotion programs, and educational material) were studied and evaluated for their effectiveness in improving good handwashing behaviors and for their potential to scale. Nine studies explored the factors that facilitated or impeded handwashing with soap. Seven studies focused on researching hand hygiene practices in a geographic area. Their interests included understanding types of handwashing practices, exploring the socio-environmental context, and gaining insights on the influence of sanitation management.

## Theoretical Domains Framework (TDF)

**Fig 2:**
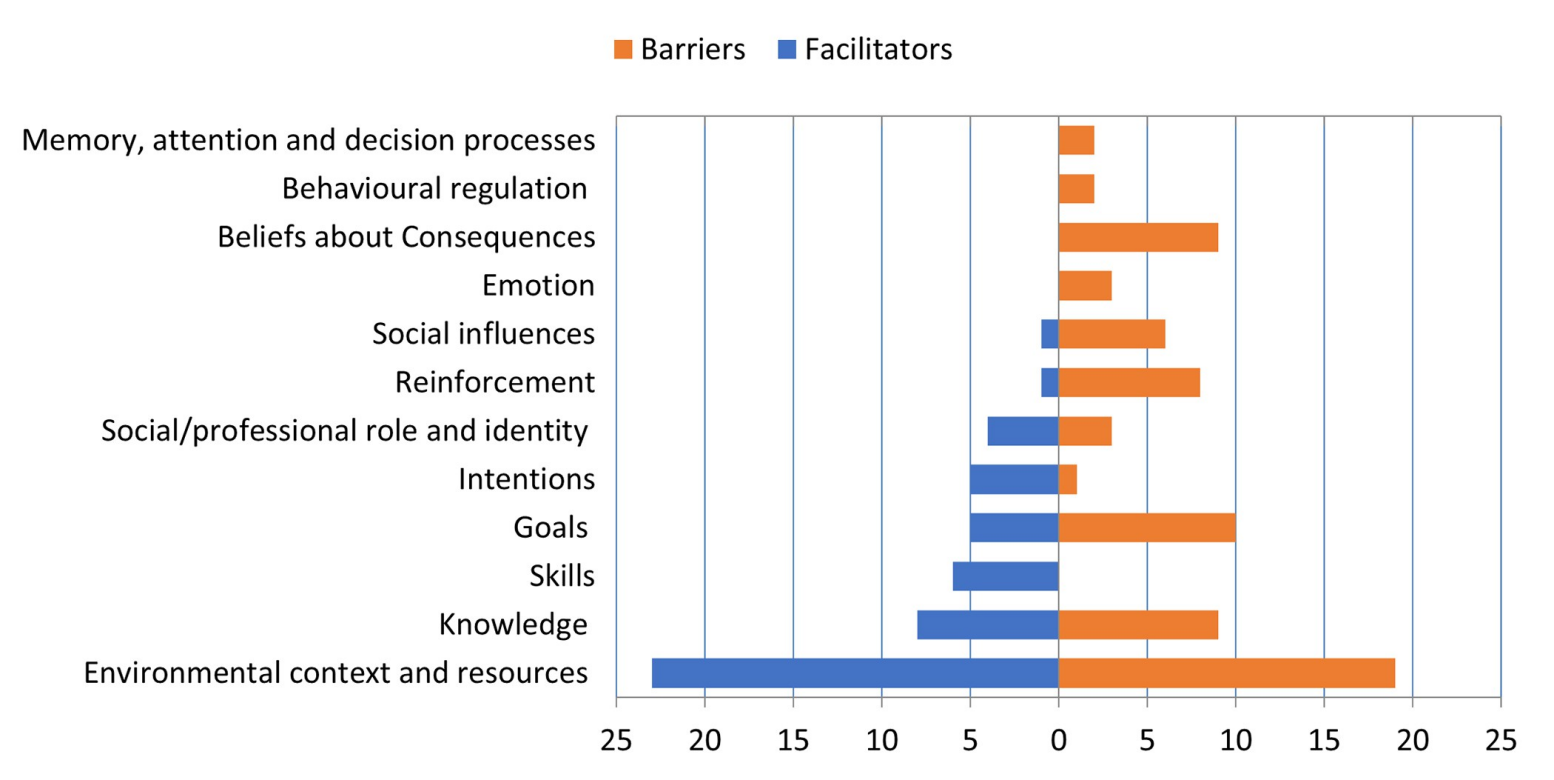


The TDF is used to identify the influences of health-related behaviors in the context of implementing evidence-based recommendations to improve health [15]. It is divided into 14 different domains that each describe an area where behavior change can be addressed when investigating implementation problems [15]. All barriers and facilitators in this study were mapped to 12 of the 14 domains of the TDF (Tables 2 and 3). Fig 2 illustrates the domain frequency for the extracted barriers and facilitators, where the bars indicate the number of articles that cited a particular domain as a barrier and/or facilitator.

**Table 2:** Barriers identified from the 46 studies categorized into domains of the Theoretical Domains Framework.

Barrier Name	Countries	Representative Excerpt	References	Theoretical Domain
Lack of available physical handwash resources (11)	Bangladesh (1); Democratic Republic of the Congo (1); Egypt (1); Kenya (1); Malawi (1); Nepal (1); Multicountry (2); South Africa (1); Tanzania (1); Uganda (1)	“One barrier for developing a self-made handwashing station was lack of available resources in the households." (p.7; Biswas, 2017) [23]	[19, 22–24, 27, 28, 30, 31, 40, 41, 61]	Environmental context and resources
High Cost of Supply and Material (9)	Australia (1); Bangladesh (1): Democratic Republic of the Congo (1); India (1); Kenya (1); Mali (1); Multicountry (1); UK (1)	“Poverty hindered buying of slippers, soap, brush, and latrine cleaning agents. Poor and ultra-poor households extensively cited poor economic condition as a barrier in practicing hygiene measures, rendering them as unsuccessful households.” (p.7; Akter, 2014) [34]	[17, 19, 22, 30, 34, 35, 41, 42, 45]	Environmental context and resources
Traditional knowledge, beliefs, behaviors (8)	Bangladesh (2); Democratic Republic of the Congo (1); Egypt (1); Myanmar (1); Mali (1); Multicountry (1)	“Some thought that if there is no visible dirt on hands, just water without soap is sufficient for hand washing.” (p.7; Akter, 2014) [34])	[17, 19, 21, 27, 29, 30, 34, 35]	Knowledge
Ineffective handwash station design (6)	Bangladesh (2); Kenya (1); Malawi (1); South Africa (1); UK (1)	“Several schools reported that one or more containers were unusable due to plastic warping from placement in the sun, broken or sand-clogged taps, or other broken parts.” (p.124; La Con, 2017) [41]	[17, 18, 22, 26, 40, 41]	Environmental context and resources
Heavy weight and long distance to handwash resources (6)	Bangladesh (1); Democratic Republic of the Congo (1); Kenya (2); Malawi (1); Multicountry (1)	"Answers from the semi-structured qualitative interviews at the final follow-up visit suggested that the top bucket could be heavy for students to fill with water and lift onto the frame, and students did not consistently make the soapy water and re-fill the container when empty.” (p.5; Wichaidit, 2019) [38]	[19, 20, 22, 34, 38, 41]	Skills
Competing priorities of household tasks and time (5)	Bangladesh (2); Democratic Republic of the Congo (1); N**/**A (1); UK (1)	"For both intervention groups mothers reported that they forgot to wash their hands due to the pressure of household chores." (p.6-7, Biswas, 2017) [23]	[16, 19, 23, 26, 29]	Goals
Inadequate household/community infrastructure (5)	Bangladesh (1); Democratic Republic of the Congo (1); Egypt (1); Multicountry (1); South Africa (1)	"Lack of a drainage system was another commonly cited barrier to washing hands in Villages 2 and 3. Households tried to use as little water as possible to prevent filling their sewage tanks, because they could not afford to empty them frequently." (p.222; Lohiniva, 2008) [27]	[17, 19, 27, 30, 43]	Environmental context and resources
Lack of time for handwashing (4)	Egypt (1); UK (1); Kenya (1); Multicountry (1)	"Many respondents talked about their lives, workload, and domestic responsibilities, like simultaneously cooking and taking care of children and animals. In this context, the necessary handwashing was considered impractical and even impossible." (p.222; Lohiniva, 2008) [27]	[26, 27, 30, 41]	Environmental context and resources
Time required to replenish water supply (3)	Democratic Republic of the Congo (1); Myanmar (1); Multicountry (1)	"Other barriers to handwashing included distances and time required to obtain water and the need to eat quickly when sharing a communal plate with family members." (p.9; Blum, 2019) [19]	[19, 21, 30]	Environmental context and resources
Unwillingness and reluctance to practice handwashing (3)	Democratic Republic of the Congo (1); Malawi (1); Vietnam (1)	“The major challenge was reluctance. The pupils felt the facilities were of no importance maybe because in their home they are not used to washing hands. I believe there is big problem in our communities of that people don’t value the importance of washing hands." (p.7; Parkinson, 2018) [22]	[19, 22, 33]	Intention
Theft of handwashing material and resources (2)	Bangladesh (1); Malawi (1)	"Theft of water buckets and soap was also identified as a significant barrier to handwashing behavior. In all three districts, school children and the wider community have been involved in stealing soap and buckets left in handwashing facilities or toilets at school, and schools struggle to replace these items regularly." (p.9; Parkinson, 2018) [22]	[19, 22]	Environmental context and resources
Handwash station damage by animals and children (2)	Bangladesh (1); Mali (1)	"Although, the main reported barrier for both intervention group categories was wastage as children frequently play with the water and soap/soapy-water which was more frequently reported by respondents from the self-made HWS group." (p.7, Biswas, 2017) [23]	[23, 35]	Environmental context and resources
Lack of responsibility ownership to maintain handwash station (2)	Bangladesh (2)	"During the focus group discussions, most of these households found preparing soapy water an additional burden over and above their other routine responsibilities, and were unwilling to prepare soapy water to share with others, or to pay for and to share responsibility for soapy water preparation." (p.506; Sultana, 2018) [18]	[7, 18]	Social/professional role and identity
Lack of time to maintain handwashing station (2)	Bangladesh (1); Mali (1)	"Several reasons are attributed to the seasonal decrease in the use of soap. Primarily, station users may be too busy to maintain and refill stations with increased workloads during this wetter cultivation period. During this season, stations are not used as much since most women and men spend the day and eat lunch in the fields." (p.284; Naughton, 2015) [35]	[34, 35]	Intention
Inaccessibility of handwashing resources due to theft (2)	Bangladesh (1); Uganda (1)	"Because of the perceived value of the provided handwashing station, household members expressed concern regarding theft during the night. This led some households to keep the stations indoors after dark hours, making it unavailable during the night to facilitate handwashing." (pg. 426; Ashraf, 2017) [20]	[20, 44]	Environmental context and resources
Prioritization of soap for uses other than handwashing (2)	Multicountry (1); Uganda (1)	"Majority of the households, 97.1% (265/312) obtained their soap from a retail shop. However, only 2.6% (7/312) reserved soap for hand washing as a high priority." (pg.12; Namara, 2020) [31]	[31, 39]	Environmental context and resources
Inadequate water supply quality (1)	South Africa (1)	"Factors relating to the water supply (temperature, cleanliness and smell) were also potential barriers to handwashing." (p.6; Burns, 2018) [40]	[40]	Environmental context and resources
Lack of time for the school management committee to support hand hygiene activity (1)	Bangladesh (1)	"When the soap runs out the teachers don’t replace it for a month. . .According to teachers and field officers, barriers to SMC activity include that members are busy as explained by a teacher at school 3, ’The SMC doesn’t have time to visit the school." (p.6-10, Chatterley, 2014) [16] *"Save the Children and government partners also provide health-related training and guidance to the SMC—a group of 12 community members and teachers that meet monthly to manage school activities according to government mandate." (p.2) [16]	[16]	Social influences
Absence of sanitation champions (1)	Bangladesh (1)	"On the other hand, teacher transfer may remove champions, as girl students from school 3 explain, ’When we were in grade 4 we had a teacher… but he transferred to another school. Since then no one talks to us about handwashing.’ To an extent, the field officers are all acting as champions, and though active field officers can be a positive influence, caution may be needed to discourage schools from relying on them." (p.11, Chatterley, 2014) [16]	[16]	Social/professional role and identity
Placement of handwashing stations (1)	Bangladesh (1)	"Placement of the handwashing station influenced the ability of the device to provide visual cues. In most households, keeping the handwashing device at the toilet was too far removed from other household activities to serve as a reminder to wash hands before cooking or eating." (p.7; Hulland, 2013) [17]	[17]	Reinforcement
Low exposure to handwashing hardware for men engaged in labor (1)	Democratic Republic of the Congo (1)	"Group discussion participants mentioned that people engaged in wage labor, who are mostly men, have less exposure to handwashing hardware and camp-based messaging, while female residents have greater contact with hygiene promoters." (p.11; Blum, 2019) [19]	[19]	Social/professional role and identity

***Note***. One sample excerpt is listed for each barrier, and the number in brackets following the name of each barrier indicates the number of citations identified.

**Table 3:** Facilitators identified from the 46 studies categorized into domains of the Theoretical Domains Framework.

Facilitator Name	Countries	Representative Excerpt	References	Theoretical Domain
Health promotion programs (10)	Bangladesh (1); Democratic Republic of the Congo (1); Ghana (1); India (2); Kenya (1); Myanmar (1); Tanzania (1); UK (1); Zambia (1)	"Overall, children were deeply affected by CLTS triggering. Most participants reported that children have changed sanitation behaviors (including using latrines and washing hands) as a direct result of CLTS. Child-centered activities, including song and dance, were frequently mentioned as important components of CLTS triggering, stimulating youth involvement and, eventually, behavior change." (p.557; Lawrence, 2016) [46]	[19–21, 24, 38, 46–48, 55, 56]	Goals
Hygiene knowledge/beliefs/values (9)	Bangladesh (1); Democratic Republic of the Congo (1); Egypt (1); Nepal (1); Multicountry (1); Tanzania (1); UK (1); United States (1)	"Knowledge of the importance of washing hands to reduce the spread of germs was high among pupils, with this being mentioned in all focus group." (p.1064; Chittleborough, 2012) [26]	[19, 23–30]	Beliefs about Consequences
Education on hand hygiene (9)	India (1); Mali (1); Myanmar (1); Tanzania (1); Thailand (1); UK (2); United States (2)	"Intensive hand washing education significantly increased self-reported frequency by 2 episodes per day, the quality of hand washing by 3 scores and improved hand washing technique." (p.4; Kaewchana, 2012) [57]	[21, 24–26, 35, 57–60]	Knowledge
Convenient location/placement of handwash supplies (8)	Bangladesh (3); Kenya (2); Myanmar (1); Mali (1); Tanzania (1)	"Participants from the arms who received a handwashing station reported that the station helped them to store water at locations convenient to the toilet and the kitchen, which facilitated handwashing." (p.425; Ashraf, 2017) [20]	[18, 20, 21, 23, 24, 35, 37, 41]	Environmental context and resources
Easy-to-use handwash station design (7)	Bangladesh (3); South Africa (1); Tanzania (1); Uganda (1)	"Following structural environment modifications that reconnected the taps in the toilet facilities and repaired the broken taps at the outside sinks, hand washing, and specifically hand washing after toilet use, increased." (p.8; Bulled, 2017) [52]	[17, 23, 24, 44, 51, 52]	Environmental context and resources
Visual cues or reminders to handwash (5)	Bangladesh (4); UK (1)	"All of the teachers mentioned reminding pupils to wash their hands, and some thought that supervision improves hand washing practices. . .Seeing other people wash their hands was thought to have a positive influence on hand washing. . .Posters were seen as helpful visual reminders to wash hands." (p.1062; Chittleborough, 2012) [26]	[17, 18, 20, 26, 52]	Reinforcement
Favourable soap quality and characteristics (4)	Democratic Republic of the Congo (1)’ Iraq (1); South Africa (1); UK (1)	"Soap was also considered preferable due to its multi-faceted uses, including washing the body, clothes and dishes, as well as it being manufactured, which signified high quality and special ingredients. . . Soap was reported to encourage handwashing, with some asserting that the lather eliminates dirty substances and conveys cleanliness. Other attributes included that soap makes hands smell good, feel light, smooth, and soft, and look clean and pretty, making the user feel good, at ease and confident." (p.8-10; Blum, 2019) [19]	[19, 26, 40, 54]	Reinforcement
Emotions that drive the need or desire to handwash (3)	India (1); Nepal (1); Multicountry (1)	"Disgust was a motivator for hygiene behaviours: hands were washed to remove “dirty things” particularly after defecation, and open defecation was regarded as dirty. Good hygiene was regarded as a way to avoid contamination of immediate and wider environments." (p.32; McMichael, 2016) [28]	[28, 30, 61]	Emotion
Affordable Cost of Supply and Material (3)	Bangladesh (3)	"Most of the respondents that participated both in the in-depth interviews and focus group discussions found the 30-g detergent used for making the soapy water agent to be affordable and allowed sharing the cost among compound members." (p.505; Sultana, 2018) [18]	[18, 23, 52]	Environmental context and resources
Availability of handwash resources (3)	Bangladesh (1); Democratic Republic of the Congo (1); South Africa (1)	"During the in-depth interviews and focus group discussions, the most commonly stated strength of the soapy water bottle was its availability as a dispenser which facilitated shared use. The bottles were reported as easily accessible and user-friendly devices that facilitated sharing the bottle for handwashing among all the respondents who participated in the study." (p.505; Sultana, 2018) [18]	[18, 19, 51]	Environmental context and resources
Handwash material provision (3)	Kenya (1); Tanzania (1); Zambia (1)	“"Beyond the physical availability of handwashing facilities, the most important facilitator to handwashing was the consistent provision of supplies (water and soap).".” (p.6; Okello, 2019) [24]	[24, 49, 50]	Environmental context and resources
Designated handwash champion (3)	Bangladesh (2); Malawi (1)	“"This study found several schools use prefects or school student leaders to motivate the students to wash their hands. Prefects are given extra responsibility to help positively influence their classmates".” (p.7; Parkinson, 2018) [22]	[16, 18, 22]	Social/professional role and identity
Social influence to handwash (3)	Democratic Republic of the Congo (1); Eastern Zambia (1); Uganda (1)	"He revealed that exemplary neighbours who actively participated in these promotional programs were a major motivator for the non-participating neighbours to also take part in these programs. This can be seen in the quote below: ’[…] if the neighbour did the correct hand washing, it motivates other households to do likewise … (Key Informant II). " (p.9-10; Namara, 2020) [31]	[19, 31, 53]	Social influences
Mothers’ role as handwashing teachers in the household (2)	Bangladesh (2)	"Mothers took the primary responsibility of promoting good handwashing practice among their family members by encouraging their children, husbands, and in-laws. Mothers reported that they taught their children how to wash hands themselves and often also assisted with the process, especially for younger children who could not independently wash their hands. Usually mothers maintained the handwashing station by refilling it when empty." (p.426; Ashraf, 2017) [20]	[17, 20]	Social/professional role and identity
Accessibility to handwash facility (2)	Nepal (1); UK (1)	"Most cited ease of access to clean water, close to their toilets and homes, as a primary motivation for handwashing. . . The findings suggest that hygiene habit formation was supported by ease of access to hardware (e.g. toilets/taps) and reinforcement of key hygiene behaviours, such as critical times for handwashing." (p.32; McMichael, 2016) [28]	[26, 28]	Environmental context and resources
Exposure to media relaying health promotional messages, knowledge, and information (2)	India (1); Kenya (1)	"The univariate analysis indicates that the number of media a respondent was exposed to and the number of media owned were strongly correlated with handwashing. There did not appear to be a threshold effect: handwashing increased in a roughly linear way with every additional item exposed to or owned." (p.1358; Schmidt, 2009) [37]	[36, 37]	Memory, attention and decision processes
Family members appointed as a champion within a household (2)	South Korea (1); Vietnam (1)	"The results indicate that the parent–child bond and the amount of time that children spend with their parents have significant impacts on children’s hand cleansing behaviors." (p.169; Song, 2012) [32]	[32, 33]	Social influences
Latrine ownership (1)	Myanmar (1)	"There is strong evidence to suggest that latrine ownership and handwashing habits are mutually related (Table 1). Fifty-four per cent of sanitary latrine owners wash hands with soap, compared with 20% among those without a latrine." (p.143; Bajracharya, 2003) [21]	[21]	Environmental context and resources
Household visit by authorities (1)	Myanmar (1)	"The household visit had a big influence in the. . .adoption of handwashing practice with soap after toileting." (p.145; Bajracharya, 2003) [21]	[21]	Social/professional role and identity
Handwash station maintenance (1)	UK (1)	"Having attractive and clean facilities available was seen to encourage hand washing." (p.1060; Chittleborough, 2012) [26]	[26]	Intentions
Designated handwash station (1)	Bangladesh (1)	"However, some participants noted that their newly acquired experience of a designated handwashing station facilitated habit formation. One participant described how her household’s frequent use of the drum changed how they felt about handwashing, “In the last few days we are becoming habituated to hand washing, and now if we don’t wash our hands then we feel bad." (p.8; Hulland, 2013) [17]	[17]	Behavioral regulation
Formation of handwashing habit (1)	UK (1)	"Teachers noted that including hand washing as part of the routine, for example, before lunch, or after doing activities such as painting, ensured that children washed their hands at key times during the day… There was evidence that hand washing as part of the daily routine was more common among younger than older pupils, and that opportunities existed to make hand washing more of a routine." (p.1060; Chittleborough, 2012) [26]	[26]	Behavioral regulation
Community sharing of a soapy water system (1)	Bangladesh (1)	"During both in-depth interviews and focus group discussions, these compound members who shared a common soapy water system with their neighboring households mentioned that it created a supportive handwashing environment as it provided an innovative alternative to bar soap and a new and easy homemade solution for handwashing, and included intensive encouragement by the community health promoters, which were effective for the routine use of the system." (p.505; Sultana, 2018) [18]	[18]	Social influences

*Note*. One sample excerpt is listed for each facilitator, and the number in brackets following the name of each facilitator indicates the number of citations identified.

### Social/Professional role and identity

A social/professional role and identity can be defined as “a coherent set of behaviors and displayed personal qualities of an individual in a social or work setting” [15]. Constructs in this domain are overlapping, such as professional identity, professional role, social identity, identity, professional boundaries, professional confidence, group identity, leadership, and organizational commitment [15].

Four out of 46 studies cited the lack of a social or professional role and identity as a barrier to hand hygiene. Three studies conducted in the community context of Bangladesh identified this as a barrier for community members to wash their hands. These studies reported that the absence of a sanitation champion in elementary schools demotivated students to wash their hands; moreover, they found that the lack of responsible ownership to maintain daily function of a shared handwash station in compounds led to quarrels and nonfunctional handwash facilities [16–18]. One study highlighted that the gender role of men in the Democratic Republic of the Congo posed a barrier to handwashing because of men’s engagement in wage labor in settings with low exposure to handwashing hardware [19].

Six of the 46 studies reported that the presence of a social or professional model facilitated handwashing practices. Two studies mentioned that mothers in Bangladesh take on the primary responsibility of promoting good hand hygiene by teaching their children how to use the handwash station [17, 20]. In Myanmar, one study mentioned that household visits by local authorities had a significant influence on the adoption of handwashing practices after defecation [21]. In a broader sense, the designation of a handwash champion in Bangladesh and Malawi could take the form of a schoolteacher or a compound manager whose objective was to engage the students and community members to foster a good hand hygiene environment by encouraging them to wash their hands at key times and replenish the soapy water supply [16, 18, 22].

### Beliefs about consequences

Beliefs about consequences have to do with the “acceptance of the truth, reality, or validity about outcomes of a behavior in a given situation” [15]. Constructs under this domain include beliefs, outcomes, outcome expectancies and their characteristics, anticipated regret, and consequents [15].

Nine out of 46 studies cited that the beliefs surrounding the consequences of poor hand hygiene were found to facilitate and influence decision-making about HWWS. Two studies mentioned mothers’ value perception, wherein food would not provide nourishment to children if the feeding hands were not washed, and they reported that convenient communal handwash stations were valued by mothers as a reminder for everyone to wash their hands [19, 23]. One study followed a hand hygiene intervention in a school in Tanzania, where value related to proper hand hygiene practice was created from knowledge of helminth infection transmission, which motivated students and teachers to wash their hands [24]. Another five studies outlined that the general knowledge that germs cause disease was the main motivator in HWWS because the participants knew that handwashing action could protect individuals’ health and prevent the transmission of disease to others [25–29]. One study found that in Nepal and Pakistan, religious beliefs about purity were an important driver for HWWS, where physical cleanliness was deeply intertwined with participants’ faith [30].

### Social influences

Social influences are defined as the “interpersonal processes that can cause individuals to change their thoughts, feelings, or behaviors” [15]. Constructs under this domain include social pressures, social norms, social comparisons, social support, group conformity, group norms, group identity, intergroup conflict, power, alienation, and modeling [15].

Six out of 46 studies mentioned that social influences served as facilitators to encourage handwashing through interactions within a social setting. Three studies mentioned that the social pressures exerted by community members motivated handwashing. It was found that conforming to hand hygiene rules in churches in the Democratic Republic of the Congo was done to maintain a good image; moreover, in Uganda, households observed to perform correct handwashing practices by neighbors influenced them to do the same [19, 31]. In addition, children reminding their mothers to wash their hands acted as a form of social influence, facilitating handwashing behavior [53]. Another study in Bangladesh found that neighboring households that shared a soapy water system produced a supportive handwashing environment, where everyone actively took responsibility for its maintenance [18]. Two other studies in Asia reported that family interactions—specifically, parent–child bonding and sibling-to-sibling interactions—facilitated engagement in handwashing behaviors [32, 33]. Another study found that the negative social influence of missing communal support was a barrier in Bangladeshi schools: the schools lacked community members and teachers who were responsible for heading good sanitation (e.g., soap provision), or those who had this responsibility were inactive in fulfilling it due to a lack of time for Save the Children and government partners to train the school management committee [16].

### Intentions

Intentions involve a “conscious decision to perform a behavior or a resolve to act in a certain way” [15]. Constructs under this domain include the stability of intentions, the stages of change model, the transtheoretical model, and stages of change [15].

One of the 46 studies reported that the intention to maintain attractive and clean handwashing stations was seen to encourage and facilitate handwashing in an elementary school in the UK [26]. Five out of 46 studies identified the intention not to wash hands as a barrier to hand hygiene. The unwillingness to practice handwashing was cited in three studies in which inhabitants of camps in the Democratic Republic of the Congo were unwilling to subscribe to hygiene mandates, and students in Malawi and Vietnam were reluctant to handwash because of the lack of support from teachers and family [19, 22, 23]. Two other studies reported that in Bangladesh and Mali, being too busy acted as a barrier to maintaining handwashing stations and washing hands because of heavy workloads in the form of household or laborious jobs [34, 35].

### Behavioral regulation

Behavioral regulation is “anything aimed at managing or changing objectively observed or measured actions” [15]. Constructs under this domain include self-monitoring, breaking habits, and action planning [15].

One of the 46 studies found that having a designated handwashing station facilitated the habit formation to wash hands before cooking and cleaning [17]. Another study noted that establishing a daily routine for young students with the opportunity to incorporate and encourage handwashing facilitated the formation of handwashing habits [26].

### Memory, attention, and decision processes

Memory, attention, and decision processes deal with “the ability to retain information, focus selectively on aspects of the environment and choose between two or more alternatives” [15]. Constructs under this domain include memory, attention, attention control, decision making, and cognitive overload/tiredness [15].

Two of the 46 studies reported that exposure to media facilitated positive handwashing behaviors. In one study, it was found that mothers in India were more likely to wash their hands with soap on key occasions when they had seen mass media content, including television advertisements and mobile phone messaging [36]. Another study conducted in Kenya concluded that viewing media that promoted proper hand hygiene was positively correlated to handwashing [37].

### Skills

A skill is defined as “an ability or proficiency acquired through practice” [15]. Constructs under this domain include skills development, competence, ability, interpersonal skills, practice, and skills assessment [15].

Six of the 46 studies found that individuals were unable to effectively procure water since they lacked the skill and capability to physically travel long distances and transport water across a long distance due to its heavy weight [19, 20, 22, 34, 38, 41].

### Environmental context and resources

The environmental context and resources encompass “any circumstance of a person’s situation or environment that discourages or encourages the development of skills and abilities, independence, social competence and adaptive behavior” [15]. Constructs under this domain include environmental stressors, resources/material resources, and person and environment interaction [15].

Twenty-two of the 46 studies identified multiple barriers to hand hygiene because of the environmental context and resources. Most prominently, 11 studies found that the lack of handwashing resources limited the ability to carry out proper hand hygiene [19, 22–24, 27, 28, 30, 31, 40, 41, 61], due to the lack of handwash station hardware and water supply and its quality, multiuse of soap for both handwashing and laundry, and soap shortages [19, 22–24, 27, 28, 30, 31, 39–41]. Nine studies found that a high cost of materials and a low budget placed financial restraints on hand hygiene [17, 19, 22, 30, 34, 35, 41, 42, 45]. Another six studies reported that an ineffective handwash station design, particularly with high tap height, low water tank capacity, low hardware durability, and frequent damage from overuse made it difficult to wash hands [17, 18, 22, 26, 40, 41].

Five studies reported that the community complex’s infrastructure was inadequate for sustaining proper hand hygiene because of inconvenient and impermanent handwash station locations, the physical isolation of households, and the water tap to household ratio in a community [17, 19, 27, 30, 43]. Four studies reported that the time required to wash hands was a deterrent to the behavior because it was a waste of time or because there were better activities to participate in [26, 27, 30, 41]. Four studies found that theft of handwashing resources (e.g., soap bars, tippy taps) prevented others from washing their hands [19, 20, 22, 44]. Another four studies found that handwashing behavior decreased as the distance between the handwashing station and the home or latrine increased [19, 22, 30, 41]. Three studies reported that the time required to retrieve water was a disincentive to washing hands [19, 21, 30]. Two studies reported that damage to the handwash station by children and animals made it difficult to keep the facility functional [23, 35], and one study reported that issues with the temperature, cleanliness, and smell of the water supply were potential barriers to handwashing [40].

Eight of the 46 studies mentioned that convenient placement/location of handwash supplies facilitated handwashing behavior, where soap placement near the handwashing station [18, 20, 21, 23, 24, 35, 37, 41] increased accessibility [26, 28]. Availability and affordability of resources for individuals promoted resource sharing and increased HWWS frequency because the resources lasted longer [18, 19, 23, 24, 49–52]. Having a simple, convenient, and easy-to-use handwash station design ensured that individuals were capable of using the station to wash their hands [17, 23, 24, 44, 51, 52]. Finally, latrine ownership was associated with positive handwashing habits [21].

### Reinforcement

Reinforcement increases “the probability of a response by arranging a dependent relationship, or contingency, between the response and a given stimulus” [15]. Constructs under this domain include rewards, incentives, punishment, consequences, contingencies, and sanctions [15].

One of the 46 studies found that the placement of handwashing stations was perceived as a barrier; the presence of a handwashing station was equivalent to a visual cue reminder to wash one’s hands, and thus, participants who lacked a handwashing station also lacked such a cue [17]. At the same time, the sight of dirt on hands, the presence of handwashing stations, the placement of soapy water bottles, and the inclusion of environmental nudges all served as visual reminders that encouraged handwashing behavior [17–20, 52]. Likewise, studies have indicated that reminders from children to parents and teachers to pupils improved handwashing rates [26]. These rates are also positively influenced by favorable soap quality and characteristics, such as a nice smell, texture, and appearance [19, 26, 40, 54].

### Goals

Goals are “mental representations of outcomes or end states that an individual wants to achieve” [15]. Constructs under this domain are goals (distal/proximal), goal priority, goal/target setting, goals (autonomous/controlled), action planning, and implementation intention [15].

Five of the 46 articles identified that conflicting goals in the form of competing household priorities and time restraints posed barriers to handwashing. For example, mothers tended to forget to wash their hands because of competing priorities [26], such as household chores [23] and ensuring family members had sufficient food to eat [19]. When water containers were used for other priorities, their use for handwashing became less convenient [29], and handwashing and sanitation observations were only sparsely included in inspections [16]; as a result, handwashing behavior was negatively affected.

Ten of the 46 articles presented health promotion programs as facilitators of handwashing behavior. Information sessions, persuasion by health promoters, handwashing stations with soapy water, handwashing messages, activities and demonstrations, and other measures increased the frequency of handwashing behavior [19–21, 24, 38, 46–48, 55], both at school and at home [56].

### Knowledge

Knowledge is “an awareness of the existence of something” [15]. Constructs under this domain include knowledge of conditions/scientific rationale procedures and task environment [15].

In 8 of the 46 articles, traditional knowledge, beliefs, and behaviors interfered with proper HWWS. The notion was identified that if there was no visible dirt on the hands, handwashing with water alone was sufficient [19, 34]; this implied a lack of understanding regarding the importance of HWWS [35]. However, even with awareness of proper handwashing techniques, participants felt it was unnecessary to wash hands every single time because they had plenty of opportunities to do so throughout the day [27]. The customs and belief systems of the individuals also conflicted with scientifically proven hygiene practices; for instance, some participants used twigs and paper to clean their hands [19, 21], whereas some believed that baby excrement was germ-free and could be handled without requiring handwashing [30]. The public would follow proper hand hygiene in humanitarian emergency contexts but would revert back to old handwashing habits once the risk of illness had dissipated [29]. Finally, since *bodnas* (i.e., water vessels) were used for anal cleaning, their multipurpose use rendered them useless for handwashing in rural and urban areas [17].

Education on hand hygiene was a key factor in facilitating handwashing practices. When individuals received handwashing education [24, 35, 37] and had seen handwashing campaigns [25], and when they understood why and how to wash their hands [26], recalled sanitation information [21], and engaged with handwashing interventions [58, 59], their handwashing knowledge, behavior, and quality improved over time [60].

### Emotion

Emotions are “a complex reaction pattern, involving experiential, behavioral, and physiological elements, by which the individual attempts to deal with a personally significant matter” [15]. Constructs under the domain of emotions are fear, anxiety, stress, depression, positive/negative affect, and burnout [15].

Emotional drivers that facilitate handwashing were described in three of the 46 articles [28, 30, 61]. One of these drivers was disgust, which pushed people to wash their hands to remove dirt [28] and odor and to distance themselves from the stigma associated with dirty hands [30]. Mothers also washed their hands and taught their children handwashing techniques because social and cultural norms dictate how mothers should behave [30]. Other drivers were nurture and affiliation, which influenced children to adopt handwashing habits because they were taught to do so by their mothers and wanted to fit in with others [30].

### Emergent themes

**Fig 3:**
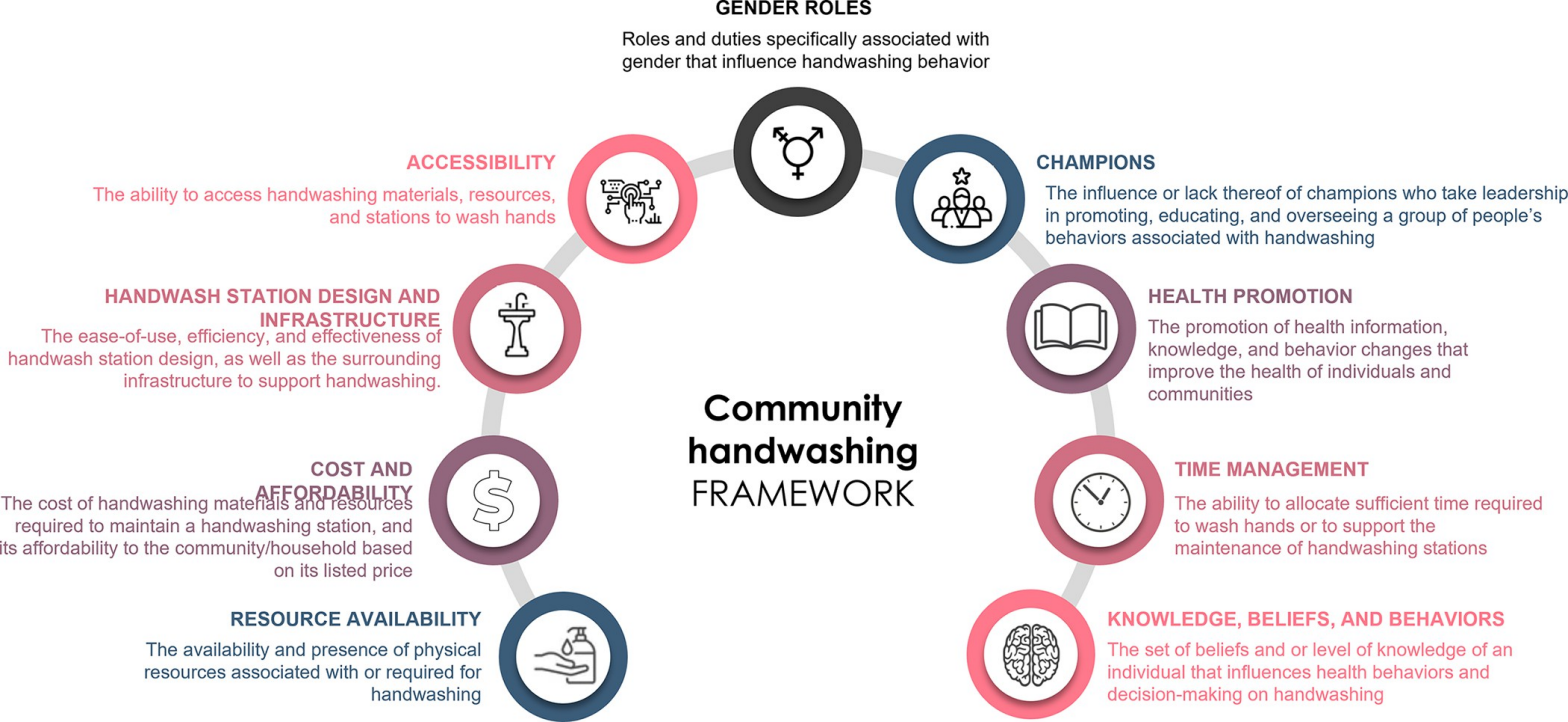


We identified nine overarching themes from the analysis of the results, including:

1. resource availability (presence of physical resources associated with or required for handwashing), 2. cost and affordability (cost of handwashing materials and resources required to maintain a handwashing station, and its affordability to the community/household based on its listed price), 3. handwash station design and infrastructure (the ease-of-use, efficiency, and effectiveness of handwash station design, as well as the surrounding infrastructure to support handwashing), 4. accessibility (the ability to access handwashing materials, resources, and stations to wash hands), 5. gender roles (roles and duties associated explicitly with gender that influence handwashing behavior), 6. champions (the influence or lack thereof of advocates who take leadership in promoting, educating, and overseeing a group of people’s behaviors associated with handwashing), 7. health promotion (the promotion of health information, knowledge, and behavior changes that improve the health of individuals and communities), 8. time management (the ability to allocate the sufficient time required to wash hands or to support the maintenance of handwashing stations), and 9. knowledge beliefs and behaviors (the set of beliefs and or level of knowledge of an individual that influences health behaviors and decision-making on handwashing) (Fig 3; S5 File).

## Discussion

This review identified multiple barriers and facilitators to implementing HWWS across 46 articles published between 2003 to 2020. Our analysis was based on 26 countries, with Bangladesh, India, and Kenya having the most representation in available studies.

Several implications revealed in this analysis can advance knowledge about enhancing HWWS. First, we note that facilitators and barriers are revealed as significant influences for most of the examined domains (Fig 2). This shows the varied nature of the factors that can influence HWWS, where 12 of the 14 TDF domains were represented. It also highlights the importance of multiple factors influencing handwashing, identifying 21 barriers and 23 facilitators, and signals that strategy to improve HWWS should depend on context i.e., removing barriers or strengthening facilitators relevant to the specific HWWS context.

Second, a few of the domains of the TDF were highly represented in the identified barriers and facilitators, specifically, *environmental context and resources*, *goals*, and *knowledge*. For example, over half the barriers fell into the environmental context and resources domain, relating to an environmentally unfriendly, resource-lacking setting, and conversely, a third of the facilitators also fell under the environmental context and resources domain, where the location, design, accessibility, availability, and affordability of handwash resources and stations supported handwashing practice. Thus, a significant opportunity to improve HWWS may be to ensure access to a desirable and conveniently located handwashing facility, with soap and water present, which is corroborated by another review on the determinants of handwashing behavior in domestic settings, where handwashing infrastructure can serve as a key facilitator [5]. We also note the importance of the goals and knowledge domains of the TDF, which were the next two frequently cited barriers and facilitators after the environmental context and resources domain, specifically around traditional knowledge, beliefs, behaviors, health promotion programs, and education on hand hygiene. Previous reviews on handwashing have discussed some of these health behavior determinants, such as knowledge and education [5], messaging with a focus on handwashing with soap [7], and reminders (written and verbal) [4] as important factors for HWWS.

Third, the identified barriers and facilitators grouped into overarching themes can serve as a framework that guides HWWS design and implementation. Practitioners can evaluate in-progress and completed HWWS projects based on whether the facilitators are missing or present and how effectively the projects have addressed barriers. The review highlights that an abundance of options for improvement may be available in light of the barriers and facilitators identified. From these findings, it is possible that in any given specific programmatic context, options can be generated. Multiple strategies to amplify psychosocial drivers to wash hands can be used to improve community handwashing [7]. For example, the emotional driver of disgust is found to motivate participation in handwashing behaviors [28, 30, 61] and can be reinforced with continuous hygiene education that teaches HWWS to prevent sickness.

Finally, the list of barriers and facilitators can be further populated from other studies not included in the review. Improvement in community-based HWWS compliance is an ongoing process and should be receptive to changes based on new data from other and future cases. In addition, the results of this review can provide contextual details to community HWWS in primary studies and cases and add to the growing evidence base on HWWS determinants.

## Limitations

This review presents several limitations that should be considered when interpreting the results. By applying search limits in the MEDLINE database (such as limiting to journal articles), partially-indexed (in-progress) content was excluded from the results. Only English-language articles were included in the review according to our inclusion criteria; this may not have captured all of the evidence for a global problem. Some studies included in the review were non-interventional and may not be as rigorous due to inherent confounding and selection bias. Across these studies, there was no standard method of data collection or analysis, and there was variance in observations, self-reported measures, handwashing frequency, and critical times for handwashing. Therefore, the findings of these studies may be too specific and not generalizable to other contexts. In addition, there were knowledge gaps in several articles that addressed HWWS without analyzing its quality or effectiveness (e.g., average handwashing time and handwashing technique). There was also a knowledge gap in situations that assessed handwashing behavior following the introduction of an intervention but did not follow up on intervention adherence over time. Since the barriers and facilitators were extracted based on interventions implemented in the short term, the evidence base and application of the results may be limited when it comes to establishing long-term goals or considering the success of future interventional strategies.

## Conclusion

This review uncovered multiple barriers and facilitators organized around a determinant framework to observe and create an in-depth, multidimensional image of a community-based hand hygiene context. Nine themes emerged from this review: resource availability, cost and affordability, handwash station design and infrastructure, accessibility, gender roles, champions, health promotion, time management, and knowledge beliefs and behaviors. The barriers and facilitators identified can be used to guide the implementation of handwashing activities and deepen the knowledge and understanding of the community contexts of hand hygiene. This has implications for strategies to be developed and implemented to overcome barriers and strengthen facilitators with the prospect of increased rates of handwashing behaviors. Stakeholders (i.e., practitioners, researchers, policymakers) can use the findings to revise, design, or evaluate new or existing projects, interventions, and policies to improve HWWS.

## Supporting information

S1 File Search strings. (DOCX)

S2 File PRISMA checklist. (DOCX)

S3 File Study characteristics. (DOCX)

S4 File Mixed-methods appraisal tool. (DOCX)

S5 File Emergent themes and their respective barriers and facilitators. (DOCX)
