# Supplementary material for: What are the barriers and facilitators to community handwashing with water and soap? A systematic review
Source: PLOS Glob Public Health. 2023 Apr 19;3(4):e0001720. doi: 10.1371/journal.pgph.0001720 (PMC10115288; doi:10.1371/journal.pgph.0001720)
Supplement: S1 File — (DOCX) [file pgph.0001720.s001.docx]

Obidimma Ezezika, Jennifer Heng, Kishif Fatima, Ayman Mohamed, and Kathryn Barrett: **What are the barriers and facilitators to community handwashing with water and soap: A systematic review**

S1 File: Search strings used for each database (OVID Medline, OVID Embase Scopus, and Web of Science)

**OVID Medline: May 15, 2020**

3,667 results

| Ovid MEDLINE: Epub Ahead of Print, In-Process & Other Non-Indexed Citations, Ovid MEDLINE® Daily and Ovid MEDLINE® <1946-Present> | | | |
| --- | --- | --- | --- |
| **#** | **Search Statement** | **Results** | **Annotation** |
| 1 | hygiene/ or hand hygiene/ or hand disinfection/ | 21849 |  |
| 2 | Soaps/ | 2507 |  |
| 3 | (hand adj3 (hygiene or wash* or clean* or soap)).tw,kf. | 7119 |  |
| 4 | (hand-wash* or handwash*).tw,kf. | 4671 |  |
| 5 | Health Behavior/ | 49395 |  |
| 6 | Health Knowledge, Attitudes, Practice/ | 110017 |  |
| 7 | health education/ or health promotion/ | 126595 |  |
| 8 | Health Policy/ | 65651 |  |
| 9 | implementation science/ | 337 |  |
| 10 | (implement* or adopt* or intervention* or program* or policy or policies or procedure* or practice* or model* or behavior* or behaviour* or routine* or knowledge or attitude* or motivation or belief* or skill* or compliance or accept* or education or promotion or efficacy or frequency or barrier* or facilitat*).tw,kf. | 9389950 |  |
| 11 | Communicable Disease Control/ | 21155 |  |
| 12 | exp Communicable Diseases/ | 35138 |  |
| 13 | epidemics/ or exp pandemics/ | 17466 |  |
| 14 | (communicable or transmission or infect* or sick* or disease* or illness* or outbreak* or epidemic* or pandemic* or pathogen* or sanitation or hygiene or prevent* or control).tw,kf. | 8601759 |  |
| 15 | (healthcare or health care or clinical or hospital* or physician* or nurse* or doctor*).tw,kf. | 5175883 |  |
| 16 | 1 or 2 or 3 or 4 | 28022 |  |
| 17 | 5 or 6 or 7 or 8 or 9 or 10 | 9473711 |  |
| 18 | 11 or 12 or 13 or 14 | 8614305 |  |
| 19 | 16 and 17 and 18 | 11071 |  |
| 20 | 19 not 15 | 5599 |  |
| 21 | limit 20 to (english language and humans and journal article) | 3807 |  |
| 22 | limit 21 to yr="1970 -Current" | 3667 |  |

**EMBASE May 15, 2020**

2,227 results

| Embase Classic+Embase <1947 to 2020 May 14> | | | |
| --- | --- | --- | --- |
| **#** | **Search Statement** | **Results** | **Annotation** |
| 1 | hand washing/ | 13620 |  |
| 2 | soap/ | 5777 |  |
| 3 | (hand adj3 (hygiene or wash* or clean* or soap)).tw,kw. | 10985 |  |
| 4 | (hand-wash* or handwash*).tw,kw. | 6260 |  |
| 5 | health behavior/ or attitude to health/ or health belief/ | 179434 |  |
| 6 | health education/ | 100679 |  |
| 7 | exp health promotion/ | 98637 |  |
| 8 | health care policy/ | 192062 |  |
| 9 | implementation science/ | 1005 |  |
| 10 | (implement* or adopt* or intervention* or program* or policy or policies or procedure* or practice* or model* or behavior* or behaviour* or routine* or knowledge or attitude* or motivation or belief* or skill* or compliance or accept* or education or promotion or efficacy or frequency or barrier* or facilitat*).tw,kw. | 12349783 |  |
| 11 | communicable disease control/ | 1077 |  |
| 12 | communicable disease/ | 29370 |  |
| 13 | epidemic/ | 119151 |  |
| 14 | exp pandemic/ | 14015 |  |
| 15 | (communicable or transmission or infect* or sick* or disease* or illness* or outbreak* or epidemic* or pandemic* or pathogen* or sanitation or hygiene or prevent* or control).tw,kw. | 11657302 |  |
| 16 | (healthcare or health care or clinical or hospital* or physician* or nurse* or doctor*).tw,kw. | 7542841 |  |
| 17 | 1 or 2 or 3 or 4 | 23180 |  |
| 18 | 5 or 6 or 7 or 8 or 9 or 10 | 12528819 |  |
| 19 | 11 or 12 or 13 or 14 or 15 | 11681818 |  |
| 20 | 17 and 18 and 19 | 12296 |  |
| 21 | 20 not 16 | 4005 |  |
| 22 | limit 21 to (human and english language and article and journal) | 2232 |  |
| 23 | limit 22 to yr="1970 -Current" | 2227 |  |

**Scopus: May 15, 2020**

3,548 results

( TITLE-ABS-KEY ( hand W/3 ( hygiene OR wash* OR clean* OR soap ) ) OR TITLE-ABS-KEY ( hand-wash* OR handwash* ) ) AND TITLE-ABS-KEY ( implement* OR adopt* OR intervention* OR program* OR policy OR policies OR procedure* OR practice* OR model* OR behavior* OR behaviour* OR routine* OR knowledge OR attitude* OR motivation OR belief* OR skill* OR compliance OR accept* OR education OR promotion OR efficacy OR frequency OR barrier* OR facilitat* ) AND TITLE-ABS-KEY ( communicable OR transmission OR infect* OR sick* OR disease* OR illness* OR outbreak* OR epidemic* OR pandemic* OR pathogen* OR sanitation OR hygiene OR prevent* OR control ) AND NOT TITLE-ABS-KEY ( healthcare OR health AND care OR clinical OR hospital* OR physician* OR nurse* OR doctor* ) AND PUBYEAR > 1969 AND ( LIMIT-TO ( SRCTYPE , "j" ) ) AND ( LIMIT-TO ( DOCTYPE , "ar" ) ) AND ( LIMIT-TO ( LANGUAGE , "English" ) )

**Web of Science: May 15, 2020**

2,101 results

You searched for: TOPIC: (((hand NEAR/3 (hygiene OR wash* OR clean* OR soap)) OR (hand-wash* OR handwash*))) AND TOPIC: ((implement* OR adopt* OR intervention* OR program* OR policy OR policies OR procedure* OR practice* OR model* OR behavior* OR behaviour* OR routine* OR knowledge OR attitude* OR motivation OR belief* OR skill* OR compliance OR accept* OR education OR promotion OR efficacy OR frequency OR barrier* OR facilitat*)) AND TOPIC: ((communicable OR transmission OR infect* OR sick* OR disease* OR illness* OR outbreak* OR epidemic* OR pandemic* OR pathogen* OR sanitation OR hygiene OR prevent* OR control)) NOT TOPIC: ((healthcare OR "health care" OR clinical OR hospital* OR physician* OR nurse* OR doctor*))

Refined by: DOCUMENT TYPES: ( ARTICLE ) AND LANGUAGES: ( ENGLISH )

Timespan: 1970-2020. Indexes: SCI-EXPANDED, SSCI, A&HCI, CPCI-S, CPCI-SSH, BKCI-S, BKCI-SSH, ESCI.

# Types of study to be included

## Inclusion criteria:

- Articles involving hand hygiene with soap and water only

- Studies involving participants of any age

- Studies involving any geographic region

- Articles written in the English language

- Articles published 1970-2020*

- Journal articles from peer-reviewed journals

- Studies that include primary research in the form of qualitative, quantitative, mixed-methods

- Studies that focus on hand hygiene and hand washing within communities, schools, general populations

- Studies that discuss barriers and facilitators to hand washing and hand hygiene

- Studies that focus on the policies, behaviours, implementation on hand hygiene and hand washing

- Studies that focus on the prevention of infectious/communicable diseases by use of hand hygiene and hand washing

*The date range parameter of 1970-2020 was chosen because the decade of 1970 is when the Centre for Disease Control and Prevention, a federal agency of a high-income country (Unites States) decided to run a nationwide investigation on the efficacy and importance of hand hygiene.

## Exclusion criteria:

- Articles that do not involve hand hygiene with soap and water

- Articles that involve hand hygiene with hand sanitizers and other similar alcohol-based products

- Articles not written in the English language

- Articles published before the year 1970

- Publication types that are not journal articles from peer-reviewed journals (e.g. books or book chapters, conference proceedings, trade journal articles, magazine articles, newspaper articles, grey literature reports

- Studies that are not primary research (systematic reviews, scoping reviews)

- Articles that focus in a healthcare, hospital, clinical setting and population

- Articles that focus on the food industry, food preparation, food sector

- Articles that do not discuss barriers and facilitators to hand washing and hand hygiene

- Studies that do not focus on the policies, behaviours, implementation on hand hygiene and hand washing

- Studies that do not focus on the prevention of infectious/communicable diseases by use of hand hygiene and hand washing
