# Supplementary material for: What are the barriers and facilitators to community handwashing with water and soap? A systematic review
Source: PLOS Glob Public Health. 2023 Apr 19;3(4):e0001720. doi: 10.1371/journal.pgph.0001720 (PMC10115288; doi:10.1371/journal.pgph.0001720)
Supplement: S3 File — (DOCX) [file pgph.0001720.s003.docx]

Obidimma Ezezika, Jennifer Heng, Kishif Fatima, Ayman Mohamed, and Kathryn Barrett: **What are the barriers and facilitators to community handwashing with water and soap? A systematic review**

**S3 File:** Study Characteristics

| Author/Year | Country | Intervention/Non-intervention | Methods | Participants (n) | Objective |
| --- | --- | --- | --- | --- | --- |
| Akter & Ali., 2014 [34] | Bangladesh | Non-intervention | In-depth interviews | 144 purposively selected women from 6 upazilas (sub-district) | To explore factors that facilitate and/or impede hygiene behavior in water, sanitation and hygiene (WASH) intervention areas using qualitative research techniques |
| Ashraf et al., 2017 [20] | Bangladesh | Intervention | Mixed methods: nonrandomized control trial, interviews, surveys, focus groups, group discussions | Household = Primary caregiver of at least 2 children <3 years of age and other family members of the household  Treatment Arms  Control = 72 Households  Soapy water promotion = 120  Soapy water promotion plus handwashing station = 103  Soapy water promotion plus handwashing station and free detergent refills = 90  Total = 385 from different villages | To test low-cost alternatives to bar soap for handwashing in rural Bangladesh and its uptake, feasibility and acceptability |
| Bajracharya, 2003 [21] | Myanmar | Non-intervention | Conducted survey from past program:  Motivated household members to construct sanitary latrines on a self-help basis, conducted orientation and planning workshops, trained for community mobilization, promoted through media | Household members | To assess of the activities pursued under National Sanitation Week (NSW) and Social Mobilization Sanitation and Hygiene (SocMob) from 1997 to 2001 |
| Biran et al., 2014 [61] | India | Intervention | Randomized control trial | 14 villages/278 households  Criteria: population between 700 to 2000 people, have state-run primary school attended by children age 8 to 13 years, have anganwadi centre (preschool) attended by children younger than 5 years | To test whether a scalable village-level intervention based on emotional drivers of behaviour, rather than knowledge, could improve handwashing behaviour in rural India |
| Biran et al., 2012 [39] | Thailand, Ethiopia, Kenya | Non-intervention | Structured observation of handwashing, questionnaire, discussions with mothers | Members of households from three long-term refugee camps | To gain a more accurate indication of actual handwashing rates and excreta disposal practices and social and economic descriptive data  To further the understanding of sanitation and hygiene in long-term camp populations |
| Biran et al., 2012 [39] | Bangladesh | Intervention | Qualitative assessments including interviews, and group discussions, quantitative observations | 80 households (caregivers) (20 selected from one village, totalling four villages)  Criteria: household has child aged 6 and 23 months being fed complimentary food | To compare effectiveness between two handwashing with soap interventions for child caregivers |
| Blum et la., 2019 [19] | Democratic Republic of Congo (DRC) | Non-intervention | Key informant interviews, rating exercises, groups discussions | Residents in camp are internally displaced persons in conflict neighborhoods  9 key informants (NGO representatives, camp residents in governing and hygiene committees, hygiene promoters),  18 female caregivers of young children under five y/o  4 groups of camp residents and hygiene promoters | To identify the motivators and barriers to handwashing in internally displaced persons camps of DRC |
| Bresee et al., 2016 [53] | Eastern Zambia | Intervention | Drawing and role-play by children, focus group discussions | Pupils (children) grades 3 to 7 of 39 female guardians from 5 schools in Lundazi District | To understand if and how children can influence their families to adopt healthy WASH behaviour in Eastern Zambia |
| Bulled et al., 1997 [51] | South Africa | Intervention | Quantitative observational data collection | Two schools: School A (400 students, 6 to 13 years old), School B (200 students, 6 to 13 years old) | To assess the physical and social environment context of handwashing among children in Limpopo, South Africa |
| Burns et al., 2018 [40] | South Africa | Intervention | Randomized control trial, surveys, two snack-tests (observation of handwashing before eating) | 203 households with children | To assess the effect of a novel handwashing intervention on children’s handwashing behaviour and outcomes |
| Burusnukul et al., 2013 [25] | S United States | Intervention | Online survey | University students, faculty, and staff | The purpose of the study was to determine the efficacy of a four-year campaign implemented by the university’s health services department |
| Chatterley et al., 2014 [16] | Bangladesh | Non-intervention | Fuzzy-set qualitative comparative analysis of 16 case schools based on in-depth qualitative data on sanitation and hygiene | Students | To improve programming and policies, and increase the effectiveness of limited development resources  To understand how and why some schools have well-managed sanitation post-intervention, while others do not |
| Chittleborough et al., 2012 [26] | UK | Intervention | A qualitative process evaluation within a cluster randomized controlled trial included pupil focus groups (n ¼ 16, aged 6–11 years), semi-structured interviews (n¼ 16 teachers) and observations of hand washing facilities | Teachers and pupils from 178 participating state primary schools | To explore factors that may influence hand washing behaviour among pupils and staff in primary schools |
| Crosby et al., 2020 [58] | India | Intervention | Pre-workshop questions, follow-up questions, observations and baseline and post-workshop assessments | Children and teachers | To investigate the effectiveness of specifically developed learning resources within socio-economic deprived areas of Ahmedabad, India |
| Dingman et al., 2020 [60] | United States | Intervention | The evaluation consisted of assessing knowledge and quality of handwashing using a linear puzzle and individual handwashing observation, respectively | 10 classrooms (20 students/classroom) between two schools | To present the results from the program with a special focus on the evaluation methods and tools used |
| Dreibelbis et al., 2016 [52] | Bangladesh | Intervention | Direct observation, provision of traditional handwashing infrastructure, and targeted handwashing nudges | 220 and 514 students from two rural primary schools | In this proof-of-concept study, we developed an inexpensive set of nudges to encourage handwashing with soap after toilet use in two primary schools in rural Bangladesh |
| Hulland et al., 2013 [17] | Bangladesh | Intervention | Interviews with participating households | 9 to 10 households per candidate, households with at least one child under the age of five  Urban site: 50 participating households  Rural site: 29 participating households | To identify a locally feasible and acceptable handwashing station that enabled frequent handwashing for two subsequent randomized trials testing the health effects of this behavior |
| Kaewchana et al., 2012 [57] | Thailand | Intervention | RCT, self-reported frequency of hand washing (FHW) and measured quality of hand washing (QHW), as well as the change of score on instruments designed to measure knowledge, attitude, and practice (KAP) of hand washing relevant to influenza | Household members aged older than 7 years living with a confirmed influenza-positive child in the household, 275 household members  HITS (Household influenza transmission study) prospectively identified a confirmed influenza pediatric patients and their households in the outpatient department at the Queen Sirikit National Institute of Child Health (QSNICH) | To assess the effect of intensive education on self-reported frequency of hand washing (FHW), measured quality of hand washing (QHW), and measured scores of knowledge, attitude, and practice (KAP) after 7 days and 90 days home-based intensive education of participants (aged >7 years) in households with a influenza-positive child |
| La Con et al., 2017 [41] | Kenya | Intervention | Observational field study of handwashing practices of children during breaks between classes on 3 different occasions  Self-administered questionnaire given to all teachers  6 focus group discussions with 44 teachers in stratified random sample of 5 schools | 28 primary schools | To evaluate the impact of the water quality and hand hygiene program on handwashing practices among students |
| Lang et al., 2012 [47] | Ghana | Intervention | Teacher training session and evaluation form, pre and post pilot program observation tool, parents and pupils focus group guidelines | Teacher training session and evaluation form, pre and post pilot program observation tool, parents and pupils focus group guidelines | To adapt, implement and evaluate an evidence-based hand hygiene public health initiative in elementary schools in Ghana |
| Lawrence et al., 2016 [46] | Zambia | Non-intervention | 67 in-depth interviews with community members and stakeholders and 24 focus group discussions with caregivers, heads of households, schoolchildren, and CLTS implementers | Community members and stakeholders | To examine the sanitation beliefs and behaviors of CLTS participants and the perceived impact of CLTS on sanitation practices in districts where CLTS implementation was recently initiated in Zambia, to inform the development of sanitation programs in the region |
| Levine et al., 2017 [45] | India | Intervention | Individual raw observations, individual reflective observations, joint reflective observations  content analysis methods including impressionistic, intuitive, interpretive, systematic, and strict text analyses | Students and teachers in primary and middle schools | To examine the rapid prototyping of a pilot school-based handwashing, safe water, and sanitation program in Chennai, India |
| Lohiniva et al., 2008 [27] | Egypt | Non-intervention | Key informant interviews, 16 focus group discussions and 21 in-depth interviews were carried out with female respondents in three different rural sites | Educated women with at least a completed primary school education (6 years);  Uneducated women who either never attended school or did not complete primary school; and  Key informants, which included school teachers, doctors at the rural health units, and people employed to empty the sewage tanks | To explore factors that influence handwashing practices in the Egyptian setting and make recommendations on how to use this information in a handwashing campaign |
| McDonald et al., 2015 [42] | Australia | Intervention | A survey questionnaire taking an ecological approach and based on the principals and constructs of the TPB (Theory of Planned Behaviour) was developed | All Aboriginal persons aged 16 years or more who were currently residing in the community were eligible to participate  865 survey questionnaires from across the six communities were completed | To report on the mass media component of The No Germs on Me (NGoM) Social Marketing Campaign to promote handwashing with soap to reduce high rates of infection among children living in remote Australian Aboriginal communities, trialling an evaluation design informed by the Theory of Planned Behaviour (TPB) |
| McMichael et al., 2016 [28] | Nepal | Non-ntervention | Qualitative data collected two and a half years after WASH intervention’s end-point: group discussions, interviews, drawings/stories of “most significant change” | 112 people participated; 57 males and 55 females. Their average age was 35 years, and they ranged from 10 to 78 years of age. Participants identified as being Dalit, Chhetri or Kham Magar | To identify the perceived drivers and constraints of sustained hygiene behaviour change, with a focus on elimination of open defecation and handwashing with soap/ash  To report on an evaluation of a water, sanitation and hygiene (WASH) intervention in mid-western Nepal |
| Musoke et al., 2018 [44] | Uganda | Intervention | Baseline household survey  Questionnaire on household water and sanitation, drinking water sources, bathroom and latrine, and rubbish disposal  Observational checklist was used to assess diferent WASH aspects including environmental sanitation, status of sanitary facilities, and state of water storage facilities  Focus group discussions  Key informant interviews | This survey involved 102 and 111 households in Kampala and Mukono, respectively | To seek solutions within the affected communities in order to improve the prevailing poor environment in slums as a deliberate effort to improve community health. |
| Naluonde et al., 2019 [49] | Zambia | Intervention | RCT, data collection and observation via mobile application | 50 government schools in Namwala District of Southern Province (25 control/25 intervention) | To identify and introduce a locally appropriate disruptive cue to improve handwashing behavior in schools in Zambia |
| Namara et al., 2020 [31] | Uganda | Non-intervention | Cross sectional study to collect quantitative and qualitative data: semi-structured questionnaire, key informant interviews, thematic content analysis | 312 refugees | To assess barriers and motivators to participation in hand washing promotion programs at household level among refugees in Rhino Camp, Arua district, Uganda |
| Naughton et al., 2015 [35] | Mali | Intervention | Measurement of soap weight versus time, surveys, 4 statistical tests (1) the Pearson’s Chi-squared test for independence; (2) independent samples t-test; (3) one-way analysis of variance (ANOVA) test; and (4) the one-way repeated measures ANOVA) | Household members | To design and implement a low cost and effective monitoring system for handwashing stations over 2 years to identify key factors in sustaining hand hygiene behaviors in rural Mali |
| Okello et al., 2019 [24] | Tanzania | Intervention | Cluster randomized trial, in-depth interviews with teachers, focus group discussions and friendship pair interviews with students | Students attending primary schools participating in the Mikono Safi Trial (Kiswahili for ‘Clean Hands. Participants comprised 16 purposively selected teachers aged between 23 and 52 years and 100 students aged 7–15 years | To qualitatively assess the effects of a multimodal school-based water, sanitation and hygiene (WASH) intervention on handwashing behaviour among primary students in North Western (NW) Tanzania |
| Parkinson et al., 2018 [22] | Malawi | Non-intervention | Study 1: Observations of primary school children HWWS behaviour  Study 2: Key Informant Interviews (KIIs) were conducted with one school administrator and one staff member from each included school to understand HWWS MOA factors affecting provision of regular access to handwashing facilities and soap to their students | Students aged 6 to 12 years, teachers, school administrators  Ten schools from each of three Malawian school districts: Nkhatabay, Salima and Mangochi  3,675 primary school children (1,900 girls; 1,775 boys) were observed | To provide formative insight, which is a key criterion for social marketing program design, to provide an evidence base for the development of social marketing programs aiming to increase school children’s HWWS behavior |
| Phaswana-Mafuya et al., 2005 [43] | South Africa | Non-intervention | The 122 participants were divided into 15 focus groups, each consisting of about eight members | 122 participants: 74 were male and 48 were female  Mean age was 40.1 years | To gain insight and understanding of factors that could motivate people to adopt safe hygienic practices |
| Ray et al., 2010 [48] | India | Intervention | An interview technique as well as observation of hand washing practices, surveys | 100 households | To evaluate a project implemented to improve service delivery for childhood diarrhea management through the public health sector. |
| Kumar et al., 1987  [74] | India | Intervention | Surveillance | Children under 6 years of age from 69 villages. | To study the hand washing practices followed in two urban slums as well as to assess and compare the status of different components of hand washing at the pre- and post-intervention phases |
| Rutter et al., 2020 [59] | UK | Intervention | Five evaluation methods: participant demographic (document analysis), handwashing frequency (counting product consumption), handwash quality (counting microbial presence), design persuasiveness (child interview study), stakeholder views (staff interview study) | Three UK schools and one museum - All four settings cater to children aged 4–11, with the partner museum also catering to younger children | To evaluate the effectiveness and efficiency of persuasive space graphics (PSG) in motivating handwashing in English primary school toilets |
| Saboori et al., 2013 [50] | Kenya | Intervention | Multiple rounds of structured observations of hand washing events after latrine use and  hand rinse samples were collected one time in a subset of schools  Interviews | 60 Kenyan primary schools  Eligibility criteria:  Previously enrolled in the SWASH+ impact study,  At least 25% of latrines in each school were rated dirty by previous SWASH+ analysis,  Distance to dry season water source was not more than 1,000 m, and  schools were located in the geographic strata of Kisumu/ Nyando or Rachuonyo | To identify effective, sustainable, and scalable strategies for improving WASH behaviors and educational outcomes for primary school children  To assess whether supplying soap to primary schools on a regular basis increased pupil hand washing and decreased Escherichia coli hand contamination |
| Sagan et al., 2019 [30] | Nepal, Pakistan, and Philippines | Non-intervention | Household surveys, structured observations, key informant interviews, focus group discussion, and behavioural trials | Mothers and children under the age of 18 affected by emergencies in different phases of crises | To determine emotional motivators and barriers to promote handwashing with soap among mothers affected by an emergency |
| Schmidt et el., 2009 [37] | Kenya | Non-intervention | Nationwide cross sectional survey in 800 households with two components:  Direct structured observation of hygiene practices at key junctures (food handling, cleaning a child after defaecation, toilet use), followed by  A structured interview addressing potential socio-economic, water access and behavioural determinants of handwashing. | 800 households | To determine to what extent structural constraints such as water access, media access and other poverty-associated factors limit the adoption of better handwashing practices |
| Song et al., 2012 [32] | South Korea | Non-intervention | Surveys | Sixth-grade students (n = 2323) and their parents (n = 2089)  11 elementary schools randomly selected from the Seoul Metropolitan Area, Korea | To bring about practical implications for public health promotion efforts by investigating specific factors influencing children’s hand hygiene  To determine the effects of the parent–child relationship on children’s handwashing behaviors  To investigate handwashing practice and thoroughness of hand cleansing among elementary school children |
| Sultana et al., 2018 [18] | Bangladesh | Intervention | 30 in-depth interviews and five focus group discussions among purposively selected compound members | 152 participating compounds | To evaluate the acceptability and feasibility of a shared handwashing intervention |
| Tidwell et al., 2019 [36] | India | Intervention | Sticker diaries, interviews, surveys | Study 1 (TV ads): (n = 756) mothers with at least one child 4–12 years old who regularly watched one hour of TV a day with a cable connection at home  Study 2 (SMS): New mothers (n = 598) and mothers of 4–7 year-old children (n = 501) | To increase the frequency of handwashing with soap with mass-scale media interventions |
| Tidwell et al., 2020 [56] | India | Intervention | Sticker diaries | Households with children aged 8–13 attending the nearby school were enrolled in the study | To examine the impacts on handwashing with soap on key public health occasions and differences in impact for school and home settings |
| Vujcic et al., 2015 [29] | N/A | Non-intervention | Key informant interviews | 12 WASH experts with field experience in multiple humanitarian emergencies working in water, sanitation, and hygiene | To examined current approaches, challenges, and knowledge gaps in relation to handwashing promotion in emergency settings |
| Watson et al., 2019 [54] | Iraq | Intervention | Structured observation | Older children between the ages of 5 and 12  80 households (40 households per study arm) | To test the hypothesis that a rapidly deployable and simple household intervention, designed to appeal primarily to the motives of play and curiosity using a modified bar of soap delivered in a fun and interactive session, can increase handwashing at key occasions by older children in a humanitarian emergency setting |
| Wichaidit et al., 2019 [38] | Kenya | Intervention | A cluster randomized trial  Semi-structured qualitative interviews  Structured observation | Thirty (n = 30) schools in Kisumu County  Eligibility criteria:  Be public  Be a day school, without boarding students  Have an existing handwashing system with soap and water  Have at least 200 students  Lack access to a piped connection providing continuous water supply (schools with intermittent water supply were eligible) | To evaluate an equipment-behavior change intervention’s effect on handwashing outcomes, and monitor functionality of the Povu Poa prototypes to identify design improvements necessary for continued high usage in institutional settings |
| Xuan et al., 2013 [33] | Vietnam | Intervention | Structured observations, interviews | Schoolteachers, children and parents from four selected schools in the study communes | To investigate responses to a teacher-centred participatory HWWS intervention in a multi-ethnic population of primary schoolchildren in northern rural Vietnam. |
| Yardley et al., 2011 [55] | UK | Intervention | Online questionnaires | 517 nonblinded adults aged over 18 years recruited through primary care  People with home Internet access and living with at least one other household member | To evaluate whether a Web-based intervention can encourage more frequent hand-washing in the home  To examine potential mediators and moderators of outcomes, as a necessary first step before testing effects of the intervention on infection rates in the PRIMIT trial (Primary care trial of a website-based Infection control intervention to Modify Influenza-like illness and respiratory infection Transmission) |
