# Supplementary material for: What are the barriers and facilitators to community handwashing with water and soap? A systematic review
Source: PLOS Glob Public Health. 2023 Apr 19;3(4):e0001720. doi: 10.1371/journal.pgph.0001720 (PMC10115288; doi:10.1371/journal.pgph.0001720)
Supplement: S4 File — (DOCX) [file pgph.0001720.s004.docx]

Obidimma Ezezika, Jennifer Heng, Kishif Fatima, Ayman Mohamed, and Kathryn Barrett: **What are the barriers and facilitators to community handwashing with water and soap? A systematic review**

**S4 File:** Mixed-methods Appraisal Tool

| Screening Questions | | | | | | | | | |  |
| --- | --- | --- | --- | --- | --- | --- | --- | --- | --- | --- |
| S1. Are there clear research questions? | | | | | | S2. Do the collected data allow to address the research questions? | | | |  |
| 1. Qualitative Studies | | | | | | | | | |  |
| 1.1. Is the qualitative approach appropriate to answer the research question? | 1.2. Are the qualitative data collection methods adequate to address the research question? | | 1.3. Are the findings adequately derived from the data? | | | 1.4. Is the interpretation of results sufficiently substantiated by data? | | 1.5. Is there coherence between qualitative data sources, collection, analysis and interpretation? | |  |
| 2. Randomized Control Trials | | | | | | | | | |  |
| 2.1. Is randomization appropriately performed? | 2.2. Are the groups comparable at baseline? | | 2.3. Are there complete outcome data? | | | 2.4. Are outcome assessors blinded to the intervention provided? | | 2.5 Did the participants adhere to the assigned intervention? | |  |
| 3. Non-Randomized Studies | | | | | | | | | |  |
| 3.1. Are the participants representative of the target population? | 3.2. Are measurements appropriate regarding both the outcome and intervention (or exposure)? | | 3.3. Are there complete outcome data? | | | 3.4. Are the confounders accounted for in the design and analysis? | | 3.5. During the study period, is the intervention administered (or exposure occurred) as intended? | |  |
| 4. Quantitative Descriptive Studies | | | | | | | | | |  |
| 4.1. Is the sampling strategy relevant to address the research question? | 4.2. Is the sample representative of the target population? | | 4.3. Are the measurements appropriate? | | | 4.4. Is the risk of nonresponse bias low? | | 4.5. Is the statistical analysis appropriate to answer the research question? | |  |
| 5. Mixed-Method Studies | | | | | | | | | |  |
| 5.1. Is there an adequate rationale for using a mixed methods design to address the research question? | 5.2. Are the different components of the study effectively integrated to answer the research question? | | 5.3. Are the outputs of the integration of qualitative and quantitative components adequately interpreted? | | | 5.4. Are divergences and inconsistencies between quantitative and qualitative results adequately addressed? | | 5.5. Do the different components of the study adhere to the quality criteria of each tradition of the methods involved? | |  |
|  | | | | | | | | | |  |
| Author/Year | | Screening Questions | | | Type of Study | | Methodological Quality Criteria | | | |
| Akter & Ali., 2014 [34] | | S1. Are there clear research questions? | | Yes | Qualitative | | 1.1 | | Yes | |
|  |  |  |  |  |  |  | 1.2 | | Yes | |
|  |  | S2. Do the collected data allow to address the research questions? | | Yes |  |  | 1.3 | | Yes | |
|  |  |  |  |  |  |  | 1.4 | | Yes | |
|  |  |  |  |  |  |  | 1.5 | | Yes | |
| Ashraf et al., 2017 [20] | | S1. | | Yes | Mixed Methods | | 5.1 | | Yes | |
|  |  |  |  |  |  |  | 5.2 | | Yes | |
|  |  | S2. | | Yes |  |  | 5.3 | | Yes | |
|  |  |  |  |  |  |  | 5.4 | | Yes | |
|  |  |  |  |  |  |  | 5.5 | | Yes | |
| Bajracharya, 2003 [21] | | S.1 | | Yes | Quantitative Descriptive | | 4.1 | | Yes | |
|  |  |  |  |  |  |  | 4.2 | | Yes | |
|  |  | S.2 | | Yes |  |  | 4.3 | | Yes | |
|  |  |  |  |  |  |  | 4.4 | | No | |
|  |  |  |  |  |  |  | 4.5 | | Yes | |
| Biran et al., 2014 [61] | | S.1 | | Yes | Quantitative | | 2.1 | | Yes | |
|  |  |  |  |  |  |  | 2.2 | | Yes | |
|  |  |  |  |  |  |  | 2.3 | | Yes | |
|  |  | S.2 | | Yes |  |  | 2.4 | | Yes | |
|  |  |  |  |  |  |  | 2.5 | | Yes | |
|  |  |  |  |  |  |  | 2.5 | | Yes | |
| Biran et al., 2012 [39] | | S.1 | | No | Mixed Methods | | 5.1 | | Yes | |
|  |  |  |  |  |  |  | 5.2 | | Yes | |
|  |  | S.2 | | Yes |  |  | 5.3 | | Yes | |
|  |  |  |  |  |  |  | 5.4 | | Yes | |
|  |  |  |  |  |  |  | 5.5 | | Yes | |
| Biswas et al., 2017 [23] | | S.1 | | Yes | Mixed Methods | | 5.1 | | Yes | |
|  |  |  |  |  |  |  | 5.2 | | Yes | |
|  |  |  |  |  |  |  | 5.3 | | Yes | |
|  |  |  |  |  |  |  | 5.4 | | Yes | |
|  |  | S.2 | | Yes |  |  | 5.5 | | Yes | |
| Blum et la., 2019 [19] | | S.1 | | Yes | Qualitative | | 1.1 | | Yes | |
|  |  |  |  |  |  |  | 1.2 | | Yes | |
|  |  |  |  |  |  |  | 1.3 | | Yes | |
|  |  |  |  |  |  |  | 1.4 | | Yes | |
|  |  | S.2 | | Yes |  |  | 1.5 | | Yes | |
| Bresee et al., 2016 [53] | | S.1 | | Yes | Qualitative | | 1.1 | | Yes | |
|  |  |  |  |  |  |  | 1.2 | | Yes | |
|  |  | S.2 | | Yes |  |  | 1.3 | | Yes | |
|  |  |  |  |  |  |  | 1.4 | | Yes | |
|  |  |  |  |  |  |  | 1.5 | | Yes | |
| Bulled et al., 1997 [51] | | S.1 | | Can’t Tell | Quantitative Descriptive | | 4.1 | | Can’t Tell | |
|  |  |  |  |  |  |  | 4.2 | | Yes | |
|  |  | S.2 | | Can’t Tell |  |  | 4.3 | | Yes | |
|  |  |  |  |  |  |  | 4.4 | | Can’t Tell | |
|  |  |  |  |  |  |  | 4.5 | | Yes | |
| Burns et al., 2018 [40] | | S.1 | | Yes | Quantitative Randomized Control Trial | | 2.1 | | Yes | |
|  |  |  |  |  |  |  | 2.2 | | Yes | |
|  |  | S.2 | | Yes |  |  | 2.3 | | Yes | |
|  |  |  |  |  |  |  | 2.4 | | No | |
|  |  |  |  |  |  |  | 2.5 | | Yes | |
| Burusnukul et al., 2013 [25] | | S.1 | | Yes | Quantitative Descriptive | | 4.1 | | Yes | |
|  |  |  |  |  |  |  | 4.2 | | Yes | |
|  |  | S.2 | | Yes |  |  | 4.3 | | Yes | |
|  |  |  |  |  |  |  | 4.4 | | Yes | |
|  |  |  |  |  |  |  | 4.5 | | Yes | |
| Chatterley et al., 2014 [16] | | S.1 | | Yes | Qualitative | | 1.1 | | Yes | |
|  |  |  |  |  |  |  | 1.2 | | Yes | |
|  |  | S.2 | | Yes |  |  | 1.3 | | Yes | |
|  |  |  |  |  |  |  | 1.4 | | Yes | |
|  |  |  |  |  |  |  | 1.5 | | Yes | |
| Chittleborough et al., 2012 [26] | | S.1 | | Yes | Qualitative | | 1.1 | | Yes | |
|  |  |  |  |  |  |  | 1.2 | | Yes | |
|  |  | S.2 | | Yes |  |  | 1.3 | | Yes | |
|  |  |  |  |  |  |  | 1.4 | | Yes | |
|  |  |  |  |  |  |  | 1.5 | | Yes | |
| Crosby et al., 2020 [58] | | S.1 | | Yes | Mixed Methods | | 5.1 | | Yes | |
|  |  |  |  |  |  |  | 5.2 | | Yes | |
|  |  | S.2 | | Yes |  |  | 5.3 | | Yes | |
|  |  |  |  |  |  |  | 5.4 | | Yes | |
|  |  |  |  |  |  |  | 5.5 | | Yes | |
| Dingman et al., 2020 [60] | | S.1 | | Yes | Quantitative Non-randomized | | 3.1 | | Yes | |
|  |  |  |  |  |  |  | 3.2 | | Yes | |
|  |  | S.2 | | Yes |  |  | 3.3 | | Yes | |
|  |  |  |  |  |  |  | 3.4 | | Yes | |
|  |  |  |  |  |  |  | 3.5 | | Yes | |
| Dreibelbis et al., 2016 [52] | | S.1 | | Yes | Quantitative Non-randomized | | 3.1 | | Yes | |
|  |  |  |  |  |  |  | 3.2 | | Yes | |
|  |  | S.2 | | Yes |  |  | 3.3 | | Yes | |
|  |  |  |  |  |  |  | 3.4 | | Yes | |
|  |  |  |  |  |  |  | 3.5 | | Yes | |
| Hulland et al., 2013 [17] | | S.1 | | Yes | Qualitative | | 1.1 | | Yes | |
|  |  |  |  |  |  |  | 1.2 | | Yes | |
|  |  | S.2 | | Yes |  |  | 1.3 | | Yes | |
|  |  |  |  |  |  |  | 1.4 | | Yes | |
|  |  |  |  |  |  |  | 1.5 | | Yes | |
| Kaewchana et al., 2012 [57] | | S.1 | | Yes | Quantitative Randomized Control Trial | | 2.1 | | Yes | |
|  |  |  |  |  |  |  | 2.2 | | Yes | |
|  |  | S.2 | | Yes |  |  | 2.3 | | Yes | |
|  |  |  |  |  |  |  | 2.4 | | No | |
|  |  |  |  |  |  |  | 2.5 | | Yes | |
| La Con et al., 2017 [41] | | S.1 | | Yes | Mixed Methods | | 5.1 | | Yes | |
|  |  |  |  |  |  |  | 5.2 | | Yes | |
|  |  | S.2 | | Yes |  |  | 5.3 | | Yes | |
|  |  |  |  |  |  |  | 5.4 | | Yes | |
|  |  |  |  |  |  |  | 5.5 | | Yes | |
| Lang et al., 2012 [47] | | S.1 | | Yes | Qualitative | | 1.1 | | Yes | |
|  |  |  |  |  |  |  | 1.2 | | Yes | |
|  |  | S.2 | | Yes |  |  | 1.3 | | Yes | |
|  |  |  |  |  |  |  | 1.4 | | Yes | |
|  |  |  |  |  |  |  | 1.5 | | Yes | |
| Lawrence et al., 2016 [46] | | S.1 | | Yes | Qualitative | | 1.1 | | Yes | |
|  |  |  |  |  |  |  | 1.2 | | Yes | |
|  |  | S.2 | | Yes |  |  | 1.3 | | Yes | |
|  |  |  |  |  |  |  | 1.4 | | Yes | |
|  |  |  |  |  |  |  | 1.5 | | Yes | |
| Levine et al., 2017 [45] | | S.1 | | Yes | Qualitative | | 1.1 | | Yes | |
|  |  | S.2 | | Yes |  |  | 1.2 | | Yes | |
|  |  |  |  |  |  |  | 1.3 | | Yes | |
|  |  |  |  |  |  |  | 1.4 | | Yes | |
|  |  |  |  |  |  |  | 1.5 | | Yes | |
| Lohiniva et al., 2008 [27] | | S.1 | | Yes | Qualitative | | 1.1 | | Yes | |
|  |  |  |  |  |  |  | 1.2 | | Yes | |
|  |  | S.2 | | Yes |  |  | 1.3 | | Yes | |
|  |  |  |  |  |  |  | 1.4 | | Yes | |
|  |  |  |  |  |  |  | 1.5 | | Yes | |
| McDonald et al., 2015 [42] | | S.1 | | Yes | Mixed Methods | | 5.1 | | Yes | |
|  |  |  |  |  |  |  | 5.2 | | Yes | |
|  |  | S.2 | | Yes |  |  | 5.3 | | Yes | |
|  |  |  |  |  |  |  | 5.4 | | Yes | |
|  |  |  |  |  |  |  | 5.5 | | Yes | |
| McMichael et al., 2016 [28] | | S.1 | | Yes | Qualitative | | 1.1 | | Yes | |
|  |  |  |  |  |  |  | 1.2 | | Yes | |
|  |  | S.2 | | Yes |  |  | 1.3 | | Yes | |
|  |  |  |  |  |  |  | 1.4 | | Yes | |
|  |  |  |  |  |  |  | 1.5 | | Yes | |
| Musoke et al., 2018 [44] | | S.1 | | Yes | Mixed Methods | | 1.1 | | Yes | |
|  |  |  |  |  |  |  | 1.2 | | Yes | |
|  |  | S.2 | | Yes |  |  | 1.3 | | Yes | |
|  |  |  |  |  |  |  | 1.4 | | Yes | |
|  |  |  |  |  |  |  | 1.5 | | Yes | |
| Naluonde et al., 2019 [49] | | S.1 | | Yes | Quantitative Randomized Control Trial | | 2.1 | | Yes | |
|  |  |  |  |  |  |  | 2.2 | | Yes | |
|  |  | S.2 | | Yes |  |  | 2.3 | | Yes | |
|  |  |  |  |  |  |  | 2.4 | | Yes | |
|  |  |  |  |  |  |  | 2.5 | | Yes | |
| Namara et al., 2020 [31] | | S.1 | | Yes | Mixed Methods | | 5.1 | | Yes | |
|  |  |  |  |  |  |  | 5.2 | | Yes | |
|  |  | S.2 | | Yes |  |  | 5.3 | | Yes | |
|  |  |  |  |  |  |  | 5.4 | | Yes | |
|  |  |  |  |  |  |  | 5.5 | | Yes | |
| Naughton et al., 2015 [35] | | S.1 | | Yes | Mixed Methods | | 5.1 | | Yes | |
|  |  |  |  |  |  |  | 5.2 | | Yes | |
|  |  | S.2 | | Yes |  |  | 5.3 | | Yes | |
|  |  |  |  |  |  |  | 5.4 | | Yes | |
|  |  |  |  |  |  |  | 5.5 | | Yes | |
| Okello et al., 2019 [24] | | S.1 | | Yes | Qualitative | | 1.1 | | Yes | |
|  |  |  |  |  |  |  | 1.2 | | Yes | |
|  |  | S.2 | | Yes |  |  | 1.3 | | Yes | |
|  |  |  |  |  |  |  | 1.4 | | Yes | |
|  |  |  |  |  |  |  | 1.5 | | Yes | |
| Parkinson et al., 2018 [22] | | S.1 | | Yes | Methods | | 5.1 | | Yes | |
|  |  |  |  |  |  |  | 5.2 | | Yes | |
|  |  | S.2 | | Yes |  |  | 5.3 | | Yes | |
|  |  |  |  |  |  |  | 5.4 | | Yes | |
|  |  |  |  |  |  |  | 5.5 | | Yes | |
| Phaswana-Mafuya et al., 2005 [43] | | S.1 | | Yes | Qualitative | | 1.1 | | Yes | |
|  |  |  |  |  |  |  | 1.2 | | Yes | |
|  |  | S.2 | | Yes |  |  | 1.3 | | Yes | |
|  |  |  |  |  |  |  | 1.4 | | Yes | |
|  |  |  |  |  |  |  | 1.5 | | Yes | |
| Ray et al., 2010 [48] | | S.1 | | Yes | Quantitative Non-randomized | | 3.1 | | Yes | |
|  |  |  |  |  |  |  | 3.2 | | Yes | |
|  |  | S.2 | | Yes |  |  | 3.3 | | Yes | |
|  |  |  |  |  |  |  | 3.4 | | Yes | |
|  |  |  |  |  |  |  | 3.5 | | Yes | |
| Rutter et al., 2020 [59] | | S.1 | | Yes | Mixed Methods | | 5.1 | | Yes | |
|  |  |  |  |  |  |  | 5.2 | | Yes | |
|  |  | S.2 | | Yes |  |  | 5.3 | | Yes | |
|  |  |  |  |  |  |  | 5.4 | | Yes | |
|  |  |  |  |  |  |  | 5.5 | | Yes | |
| Saboori et al., 2013 [50] | | S.1 | | Yes | Quantitative Randomized Control Trial | | 2.1 | | Yes | |
|  |  |  |  |  |  |  | 2.2 | | Yes | |
|  |  | S.2 | | Yes |  |  | 2.3 | | Yes | |
|  |  |  |  |  |  |  | 2.4 | | No | |
|  |  |  |  |  |  |  | 2.5 | | Yes | |
| Sagan et al., 2019 [30] | | S.1 | | Yes | Mixed Methods | | 5.1 | | Yes | |
|  |  |  |  |  |  |  | 5.2 | | Yes | |
|  |  | S.2 | | Yes |  |  | 5.3 | | Yes | |
|  |  |  |  |  |  |  | 5.4 | | Yes | |
|  |  |  |  |  |  |  | 5.5 | | Yes | |
| Schmidt et el., 2009 [37] | | S.1 | | Yes | Quantitative Non-randomized | | 3.1 | | Yes | |
|  |  |  |  |  |  |  | 3.2 | | Yes | |
|  |  | S.2 | | Yes |  |  | 3.3 | | Yes | |
|  |  |  |  |  |  |  | 3.4 | | Yes | |
|  |  |  |  |  |  |  | 3.5 | | Yes | |
| Song et al., 2012 [32] | | S.1 | | Yes | Quantitative Descriptive | | 4.1 | | Yes | |
|  |  |  |  |  |  |  | 4.2 | | Yes | |
|  |  | S.2 | | Yes |  |  | 4.3 | | Yes | |
|  |  |  |  |  |  |  | 4.4 | | Yes | |
|  |  |  |  |  |  |  | 4.5 | | Yes | |
| Sultana et al., 2018 [18] | | S.1 | | Yes | Qualitative | | 1.1 | | Yes | |
|  |  |  |  |  |  |  | 1.2 | | Yes | |
|  |  | S.2 | | Yes |  |  | 1.3 | | Yes | |
|  |  |  |  |  |  |  | 1.4 | | Yes | |
|  |  |  |  |  |  |  | 1.5 | | Yes | |
| Tidwell et al., 2019 [36] | | S.1 | | Yes | Quantitative Randomized Control Trial | | 4.1 | | Yes | |
|  |  |  |  |  |  |  | 4.2 | | Yes | |
|  |  | S.2 | | Yes |  |  | 4.3 | | Yes | |
|  |  |  |  |  |  |  | 4.4 | | No | |
|  |  |  |  |  |  |  | 4.5 | | Yes | |
| Tidwell et al., 2020 [56] | | S.1 | | Yes | Quantitative Randomized Control Trial | | 4.1 | | Yes | |
|  |  |  |  |  |  |  | 4.2 | | Yes | |
|  |  | S.2 | | Yes |  |  | 4.3 | | Yes | |
|  |  |  |  |  |  |  | 4.4 | | No | |
|  |  |  |  |  |  |  | 4.5 | | Yes | |
| Vujcic et al., 2015 [29] | | S.1 | | Yes | Qualitative | | 1.1 | | Yes | |
|  |  |  |  |  |  |  | 1.2 | | Yes | |
|  |  | S.2 | | Yes |  |  | 1.3 | | Yes | |
|  |  |  |  |  |  |  | 1.4 | | Yes | |
|  |  |  |  |  |  |  | 2.5 | | Yes | |
| Watson et al., 2019 [54] | | S.1 | | Yes | Quantitative Randomized Control Trial | | 2.1 | | Yes | |
|  |  |  |  |  |  |  | 2.2 | | Yes | |
|  |  | S.2 | | Yes |  |  | 2.3 | | Yes | |
|  |  |  |  |  |  |  | 2.4 | | No | |
|  |  |  |  |  |  |  | 2.5 | | Yes | |
| Wichaidit et al., 2019 [38] | | S.1 | | Yes | Quantitative Randomized Control Trial | | 2.1 | | Yes | |
|  |  |  |  |  |  |  | 2.2 | | Yes | |
|  |  | S.2 | | Yes |  |  | 2.3 | | Yes | |
|  |  |  |  |  |  |  | 2.4 | | No | |
|  |  |  |  |  |  |  | 2.5 | | Yes | |
| Xuan et al., 2013 [33] | | S.1 | | Yes | Qualitative | | 1.1 | | Yes | |
|  |  |  |  |  |  |  | 1.2 | | Yes | |
|  |  | S.2 | | Yes |  |  | 1.3 | | Yes | |
|  |  |  |  |  |  |  | 1.4 | | Yes | |
|  |  |  |  |  |  |  | 1.5 | | Yes | |
| Yardley et al., 2011 [55] | | S.1 | | Yes | Quantitative Randomized Control Trial | | 2.1 | | Yes | |
|  |  |  |  |  |  |  | 2.2 | | Yes | |
|  |  | S.2 | | Yes |  |  | 2.3 | | Yes | |
|  |  |  |  |  |  |  | 2.4 | | No | |
|  |  |  |  |  |  |  | 2.5 | | Yes | |
