# Supplementary material for: What are the barriers and facilitators to community handwashing with water and soap? A systematic review
Source: PLOS Glob Public Health. 2023 Apr 19;3(4):e0001720. doi: 10.1371/journal.pgph.0001720 (PMC10115288; doi:10.1371/journal.pgph.0001720)
Supplement: S5 File — (DOCX) [file pgph.0001720.s005.docx]

Obidimma Ezezika, Jennifer Heng, Kishif Fatima, Ayman Mohamed, and Kathryn Barrett: **What are the barriers and facilitators to community handwashing with water and soap: A systematic review**

S5 File: Themes of barriers and facilitators

| Themes | Description | Barriers | Facilitators |
| --- | --- | --- | --- |
| Resource Availability | The availability and presence of physical resources associated with or required for handwashing | - Lack of available physical handwash resources (11) - Theft of handwashing material and resources (2) | - Availability of handwash resources (3) - Handwash material provision (3) - Latrine ownership (1) |
| Cost and Affordability | The cost of handwashing materials and resources required to maintain a handwashing station, and its affordability to the community/household based on its listed price | - High cost of supply and material (9) - Prioritization of soap for uses other than handwashing (2) | - Affordable cost of supply and material (3) |
| Handwash station design and infrastructure | The ease-of-use, efficiency, and effectiveness of handwash station design, as well as the surrounding infrastructure to support handwashing | - Ineffective handwash station design (6) - Inadequate household/community infrastructure (5) - Handwash station damage by animals and children (2) - Inadequate water supply quality (1) - Placement of handwashing stations (1) | - Convenient location/placement of handwash supplies (8) - Easy-to-use handwash station design (7) - Visual cues or reminders to handwash (5) - Favourable soap quality and characteristics (4) - Designated handwash station (1) - Handwash station maintenance (1) |
| Accessibility | The ability to access handwashing materials, resources, and stations to wash hands. . | - Heavy weight and long distance to handwash resources (6) - Inaccessibility of handwashing resources due to theft (2) | - Accessibility to handwash facility (2) |
| Gender Roles | Roles and duties specifically associated with gender that influence handwashing behavior | - Low exposure to handwashing hardware for men engaged in labor (1) | - Mothers’ role as handwashing teachers in the household (2) |
| Champions | The influence or lack thereof of champions who take leadership in promoting, educating, and overseeing a group of people’s behaviors associated with handwashing | - Absence of sanitation champions (1) | - Designated handwash champion (3) - Family members appointed as a champion within a household (2) |
| Health Promotion | The promotion of health information, knowledge, and behavior changes that improve the health of individuals and communities | - None | - Health promotion programs (10) - Education on hand hygiene (9) - Exposure to media relaying health promotional messages, knowledge, and information (2) - Household visit by authorities (1) - Community sharing of a soapy water system (1) |
| Time Management | The ability to allocate sufficient time required to wash hands or to support the maintenance of handwashing stations | - Competing priorities of household tasks and time (5) - Lack of time for hand washing (4) - Time required to replenish water supply (3) - Lack of time to maintain handwashing station (2) - Lack of time for the school management committee to support hand hygiene activity (1) | - None |
| Knowledge, Beliefs, and Behaviors | The set of beliefs and or level of knowledge of an individual that influences health behaviors and decision-making on handwashing | - Traditional knowledge, beliefs, behaviors (8) - Unwillingness and reluctance to practice handwashing (3) - Lack of responsibility ownership to maintain hand wash station (2) | - Hygiene knowledge/beliefs/values (9) - Emotions that drive the need or desire to handwash (3) - Social influence to handwash (3) - Formation of handwashing habit (1) |

***Note***. The numbers in bracket indicate the number of participants that mentioned each barrier or facilitator.
